# Heterogeneity of treatment effect of interferon-β1b and lopinavir–ritonavir in patients with Middle East respiratory syndrome by cytokine levels

**DOI:** 10.1038/s41598-022-22742-8

**Published:** 2022-10-28

**Authors:** Yaseen M. Arabi, Ayed Y. Asiri, Abdullah M. Assiri, Mashan L. Abdullah, Haya A. Aljami, Hanan H. Balkhy, Majed Al Jeraisy, Yasser Mandourah, Sameera AlJohani, Shmeylan Al Harbi, Hani A. Aziz Jokhdar, Ahmad M. Deeb, Ziad A. Memish, Jesna Jose, Sameeh Ghazal, Sarah Al Faraj, Ghaleb A. Al Mekhlafi, Nisreen Murad Sherbeeni, Fatehi Elnour Elzein, Frederick G. Hayden, Robert A. Fowler, Badriah M. AlMutairi, Abdulaziz Al-Dawood, Naif Khalaf Alharbi

**Affiliations:** 1 Intensive Care Department, King Abdulaziz Medical City, Ministry of National Guard Health Affairs, College of Medicine, King Saud Bin Abdulaziz University for Health Sciences, King Abdullah International Medical Research Center, Riyadh, Kingdom of Saudi Arabia; 2 Intensive Care Department, Prince Mohammed Bin Abdulaziz Hospital, Riyadh, Kingdom of Saudi Arabia; 3 Infection Prevention and Control, Preventive Health, Ministry of Health, Riyadh, Kingdom of Saudi Arabia; 4 Experimental Medicine Department, King Abdullah International Medical Research Center, King Saud Bin Abdulaziz University for Health Sciences, Riyadh, Kingdom of Saudi Arabia; 5 King Abdullah International Medical Research Center, King Saud Bin Abdulaziz University for Health Sciences, Riyadh, Kingdom of Saudi Arabia; 6 Antimicrobial Resistance, World Health Organization, Geneva, Switzerland; 7 College of Pharmacy, King Saud Bin Abdulaziz University for Health Sciences, King Abdullah International Medical Research Center, Pharmaceutical Care Department, King Abdulaziz Medical City, Ministry of National Guard Health Affairs, Riyadh, Kingdom of Saudi Arabia; 8 Military Medical Services, Ministry of Defense, Prince Sultan Military Medical City, Riyadh, Kingdom of Saudi Arabia; 9 Department of Pathology and Laboratory Medicine, King Abdulaziz Medical City, Ministry of National Guard Health Affairs, College of Medicine, King Saud Bin Abdulaziz University for Health Sciences, King Abdullah International Medical Research Center, Riyadh, Kingdom of Saudi Arabia; 10 Deputyship for Public Health, Ministry of Health, Riyadh, Kingdom of Saudi Arabia; 11 King Abdulaziz Medical City, Ministry of National Guard Health Affairs, College of Medicine, King Saud Bin Abdulaziz University for Health Sciences, King Abdullah International Medical Research Center, Riyadh, Kingdom of Saudi Arabia; 12 Prince Mohammed Bin Abdulaziz Hospital, Ministry of Health, College of Medicine, Alfaisal University, Riyadh, Kingdom of Saudi Arabia; 13 Hubert Department of Global Health, Rollins School of Public Health, Emory University, Atlanta, GA USA; 14 Department Biostatistics and Bioinformatics, King Abdullah International Medical Research Center, King Saud Bin Abdulaziz University for Health Sciences, Riyadh, Kingdom of Saudi Arabia; 15 Prince Mohammed Bin Abdulaziz Hospital, Riyadh, Kingdom of Saudi Arabia; 16 Intensive Care Department, King Salman bin Abdulaziz Medical City, Madinah, Kingdom of Saudi Arabia; 17 Infectious Diseases Division, Prince Sultan Military Medical City, Riyadh, Kingdom of Saudi Arabia; 18 Division of Infectious Diseases and International Health, Department of Medicine, University of Virginia School of Medicine, Charlottesville, VA USA; 19 Institute of Health Policy Management and Evaluation, University of Toronto, Department of Critical Care Medicine and Department of Medicine, Sunnybrook Hospital, Toronto, Canada; 20 Intensive Care Department, King Abdulaziz Medical City, ICU 1425, P.O. Box 22490, Riyadh, 11426 Kingdom of Saudi Arabia

**Keywords:** Cytokines, Infectious diseases

## Abstract

Animal and human data indicate variable effects of interferons in treating coronavirus infections according to inflammatory status and timing of therapy. In this sub-study of the MIRACLE trial (MERS-CoV Infection Treated with a Combination of Lopinavir–Ritonavir and Interferon β-1b), we evaluated the heterogeneity of treatment effect of interferon-β1b and lopinavir–ritonavir versus placebo among hospitalized patients with MERS on 90-day mortality, according to cytokine levels and timing of therapy. We measured plasma levels of 17 cytokines at enrollment and tested the treatment effect on 90-day mortality according to cytokine levels (higher versus lower levels using the upper tertile (67%) as a cutoff point) and time to treatment (≤ 7 days versus > 7 days of symptom onset) using interaction tests. Among 70 included patients, 32 received interferon-β1b and lopinavir–ritonavir and 38 received placebo. Interferon-β1b and lopinavir–ritonavir reduced mortality in patients with lower IL-2, IL-8 and IL-13 plasma concentrations but not in patients with higher levels (p-value for interaction = 0.09, 0.07, and 0.05, respectively) and with early but not late therapy (p = 0.002). There was no statistically significant heterogeneity of treatment effect according to other cytokine levels. Further work is needed to evaluate whether the assessment of inflammatory status can help in identifying patients with MERS who may benefit from interferon-β1b and lopinavir–ritonavir.

Trial registration: This is a sub-study of the MIRACLE trial (ClinicalTrials.gov number, NCT02845843).

## Introduction

Middle East respiratory syndrome (MERS) is a viral respiratory disease caused by the Middle East respiratory syndrome coronavirus (MERS-CoV). MERS is often associated with severe respiratory and multi-organ failure, with a case fatality rate of 35%^1^. MERS-CoV continues to circulate in the Middle East among dromedary camels and to cause human infections; therefore, it remains public health threat^2,3^. Studies have demonstrated that critically ill patients with MERS generally mount a pro-inflammatory response characterized by elevated blood concentrations of several cytokines compared to healthy control^4^. Interestingly, there is a spectrum of the pro-inflammatory response, with approximately one-third of patients manifesting a relative hyperinflammatory sub-phenotype and two-thirds a relative hypoinflammatory one^4^. In addition, animal and human data indicate heterogeneous treatment effects of interferons according to the duration of coronavirus infections, including MERS and severe acute respiratory syndrome (SARS), such that interferon may be effective with early but not late therapy^5,6^.

The MIRACLE trial (MERS-CoV Infection Treated with a Combination of Lopinavir–Ritonavir and Interferon β-1b trial) was a randomized double-blind, placebo-controlled trial that investigated the efficacy of a combined treatment composing recombinant interferon-β1b and lopinavir–ritonavir, in comparison with placebo on 90-day all-cause mortality in hospitalized patients with laboratory-confirmed MERS^7–9^. The study found that combined treatment resulted in lower 90-day mortality in hospitalized patients with laboratory-confirmed MERS. The treatment effect was observed in patients treated within 7 days of symptom onset, in whom an approximate 80% relative reduction in mortality was found. In contrast, later initiation of therapy did not impact mortality^7–9^. In this sub-study of the MIRACLE trial^9^, we evaluated the heterogeneity of treatment effect of interferon-β1b and lopinavir–ritonavir on 90-day mortality of hospitalized patients with MERS according to cytokine levels and the time from symptom onset to treatment.

## Results

### Patient characteristics and clinical data

Seventy patients were enrolled in the current study, 32 of whom received the intervention and 38 received placebo. The two groups were similar in baseline characteristics, including age, sex, Acute Physiology and Chronic Health Evaluation (APACHE) II scores, organ support and key laboratory findings at baseline. At the time of enrollment, 13/32 (40.6%) in the intervention group 17/38 (44.7%) in the placebo group were receiving mechanical ventilation (Table 1). The median time from onset of symptoms to enrollment was similar in both groups (median 7.5 [IQR 5.0, 11.0] days compared to 8.0 [IQR 5.0, 12.0] days), respectively. Patients in the intervention group received a median of 25 (IQR 14, 28) doses of lopinavir–ritonavir compared to 26 (IQR 11, 28) placebo doses for the placebo group. Patients in the intervention group received a median of 7 (IQR 5, 7) doses of interferon-β1b in comparison to 7 (IQR 4, 7) placebo doses in the placebo group (Table S1). Co-interventions during the hospitalization, including vasopressor therapy, mechanical ventilation and renal replacement therapy, were similar in the intervention and placebo groups (Table S1). Of patients in the intervention group 10/32 patients (31.3%) died within 90 days compared to 20/38 patients (52.6%), relative risk 0.59 (95% confidence interval 0.33, 1.08, p = 0.09). Other clinical outcomes are reported in Table 2. Of note, patients in the intervention group had more days alive and outside the ICU than the placebo group (median 12.5 days, IQR 0.0, 28.0, compared to 0.0 days, IQR 0.0, 17.0, p 0.005), and had a shorter time to clearance of MERS-CoV RNA (median 15.0 days, IQR 9.0, 21.0, compared to 26.0 days, IQR 14.0, 49.0, p 0.0095).

**Table 1:** Baseline characteristics and plasma cytokine concentrations at enrollment for the intervention group, placebo group on study Day 1, and healthy controls. All cytokine levels were calculated based on mean fluorescent intensity and reported in pg/mL and are reported as medians (Interquartile range). p values for the comparisons between the intervention group and healthy controls and between placebo group and healthy controls are reported in Figure S2.

Variable	Intervention group (N = 32)	Placebo group (N = 38)	p value	Healthy control (N = 15)
Age (Years)—median (IQR)	61.0 (48.5, 68.0)	54.5 (44.0, 67.0)	0.49	
Male sex—no. (%)	27 (84.4)	32 (84.2)	0.98	
Body mass index(kg/m^2^)^†^—mean ± SD	27.8 ± 5.37	26.5 ± 5.62	0.33*	
Community-acquired acquisition—no. (%)	23 (71.9)	28 (73.7)	0.87	
Nosocomial acquisition—no. (%)	9 (28.1)	10 (26.3)		
Co-infection—no. (%)	5 (15.6)	6 (15.8)	0.98	
APACHE II^‡^—mean ± SD	19.9 ± 9.44	22.2 ± 10.10	0.34*	
SOFA score, median (IQR)	6.5 (3.0, 9.0)	7.0 (4.0, 10.0)	0.26	
**Comorbidities**—no. (%)				
Any chronic comorbidity	28 (87.5)	32 (84.2)	0.75^^	
Chronic cardiac disease	9 (28.1)	10 (26.3)	0.87	
Chronic pulmonary disease	2 (6.3)	1 (2.6)	0.59^^	
Chronic renal disease	7 (21.9)	13 (34.2)	0.26	
Diabetes with chronic complications	16 (50.0)	13 (34.2)	0.18	
**Location at time of randomization**—no. (%)				
Ward	12 (37.5)	10 (26.3)	0.32	
ICU	20 (62.5)	28 (73.7)		
**Randomization Stratum**—no. (%)				
Mechanically ventilated	13 (40.6)	17 (44.7)	0.73	
Not mechanically ventilated	19 (59.4)	21 (55.3)		
Renal replacement therapy—no. (%)	9 (28.1)	13 (34.2)	0.58	
Vasopressor—no. (%)	4 (12.5)	10 (26.3)	0.15	
Neuromuscular blockade—no. (%)	6 ( 18.8 )	11 ( 28.9 )	0.32	
Corticosteroids—no. (%)	11 (34.4)	12 (31.6)	0.80	
Platelets ×10^9^/L—median (IQR)	184.0 (147.0, 240.5)	176.5 (136.0, 218.0)	0.59	
White blood cell count ×10^9^/L—median (IQR)	6.0 (4.8, 9.0)	7.0 (4.2, 9.1)	0.94	
Lymphocyte count ×10^9^/L—median (IQR)	1.0 (0.6, 1.3)	0.8 (0.5, 1.2)	0.44	
Aspartate transaminase, units/L—median (IQR)	64.0 (38.0, 80.0)	88.0 (49.0, 114.0)	0.09	
Alanine aminotransferase, units/L—median (IQR)	40.0 (28.0, 69.0)	35.0 (21.0, 76.0)	0.81	
Bilirubin level, µmol l/L—median (IQR)	9.3 (6.1, 12.1)	9.0 (6.0, 16.6)	0.72	
Serum amylase, units/L—median (IQR)	73.0 (48.0, 99.0)	53.5 (36.0, 110.0)	0.54	
Creatinine, µmol /L—median (IQR)	109.5 (71.2, 276.5)	104.1 (67.0, 334.5)	0.99	
**Cytokines**—median (IQR)				
G-CSF	30.9 (15.5, 177.8)	28.2 (8.0, 262.5)	0.43	55.4 (18.2, 155.9)
GM-CSF	4.5 (2.1, 10.1)	2.8 (1.2, 8.3)	0.39	7.4 (5.8, 19.7)
IFN-γ	78.4 (48.1, 328.7)	109.9 (44.6, 208.6)	0.75	67.9 (36.2, 128.7)
IL-1β	3.4 (1.6, 5.1)	2.6 (1.5, 5.5)	0.93	0.9 (0.5, 1.9)
IL-2	16.6 (7.0, 31.8)	14.3 (3.8, 26.2)	0.62	15.9 (3.4, 18.9)
IL-4	1.3 (0.5, 2.7)	1.9 (0.9, 3.4)	0.46	1.3 (0.9, 1.8)
IL-5	31.7 (6.2, 74.9)	34.4 (4.7, 78.9)	0.91	37.7 (18.4, 68.6)
IL-6	124.1 (59.6, 211.3)	130.4 (83.8, 322.5)	0.35	13.2 (7.3, 18.9)
IL-7	5.2 (2.9, 11.3)	5.7 (3.9, 9.7)	0.86	5.9 (4.5, 6.2)
IL-8	177.8 (86.8, 308.6)	191.8 (134.8, 301.5)	0.45	66.5 (48.7, 86.9)
IL-10	63.4 (28.5, 126.6)	60.2 (34.6, 107.0)	0.76	30.8 (17.7, 40.0)
IL-12(P70)	4.5 (2.9, 6.4)	4.4 (2.6, 8.2)	0.91	5.4 (2.9, 6.2)
IL-13	2.3 (0.7, 8.4)	1.4 (0.6, 7.7)	0.51	2.1 (0.6, 7.1)
IL-17	6.8 (2.2, 20.8)	7.8 (2.5, 16.9)	0.96	8.7 (6.7, 11.4)
MCP-1	553.7 (292.9, 1007.9)	604.3 (300.2, 1034.5)	0.77	244.2 (146.3, 359.7)
MIP-1β	87.3 (46.5, 155.9)	66.1 (53.5, 107.0)	0.29	69.5 (45.5, 92.8)
TNF-α	187.2 (119.3, 365.5)	244.4 (147.5, 341.9)	0.74	150.2 (106.7, 197.8)

For categorical variables, chi-square test was used to calculate p values except for p values labeled with ^^ indicating the use of Fisher exact test. For continuous variables, Mann–Whitney U test was used to calculate p value except for p values labeled with * indicating the use of t test.

APACHE II: Acute Physiology and Chronic Health Evaluation II, SOFA: Sequential Organ Failure Assessment score, ICU = intensive care unit, IQR interquartile range. G-CSF: granulocyte-colony stimulating factor; GM-CSF: granulocyte–macrophage colony-stimulating factor; IFN: interferon; IL: interleukin; MCP: Monocyte chemo-attractant protein; MIP: Macrophage inflammatory protein; TNF: tumor necrosis factor.

^†^The body mass index is the weight in kilograms divided by the square of the height in meters.

^‡^Scores on the Acute Physiology and Chronic Health Evaluation (APACHE) II range from 0 to 71, with higher scores indicating more severe disease and higher risk of death.

**Table 2:** Outcomes of hospitalized MERS patients included in this study.

Variable	Intervention group (N = 32)	Placebo group (N = 38)	Relative risk (95% confidence interval)	p value
**Death from any cause**—no./total no. (%)**				
At 90 Days	10 (31.3)	20 (52.6)	0.59 (0.33, 1.08)	0.09
At 28 days	8 (25.0)	14 (36.8)	0.68 (0.33, 1.41)	0.30
During ICU stay	10 (31.3)	19 (50.0)	0.63 (0.34, 1.14)	0.13
During hospital stay	11 (34.4)	20 (52.6)	0.65 (0.37, 1.15)	0.14
Median no. of days free from supplemental oxygen (IQR)^†^	6.5 (0.0, 20.5)	0.0 (0.0, 17.0)		0.09
Median no. of days free from invasive or non-invasive mechanical ventilation (IQR)^†^	20.0 (0.0, 28.0)	1.0 (0.0, 24.0)		0.07
Median no. of days free from renal replacement therapy (IQR)^†^	26.0 (3.5, 28.0)	14.5 (0.0, 28.0)		0.23
Median no. of days free from vasopressors (IQR)^†^	27.5 (2.5, 28.0)	19.0 (0.0, 28.0)		0.15
Median no. of days free from organ support (IQR)^†^	14.5 (0.0, 28.0)	0.0 (0.0, 19.0)		0.07
Median no. of days outside the ICU (IQR)^†^	12.5 (0.0, 28.0)	0.0 (0.0, 17.0)		0.005
**Virologic outcomes**				
Median days to MERS-CoV RNA clearance (IQR) ##	15.0 (9.0, 21.0)	26.0 (14.0, 49.0)		0.0095
Median days to MERS-CoV RNA clearance among 90-d survivors (IQR)	11.0 (8.0, 17.0)	20.0 (10.0, 26.0)		0.03

Calculations of days free from supplemental oxygen, renal replacement therapy, mechanical ventilation, vasopressors, extracorporeal circulation support-, organ support and days outside the ICU were based on 28 days of observation.

For categorical variables, chi-square test was used to calculate p values. For continuous variables, Mann–Whitney U test was used to calculate p value. ##Days to clearance of MERS-CoV RNA were censored by death or hospital discharge.

### Baseline cytokine levels

**Figure 1:**
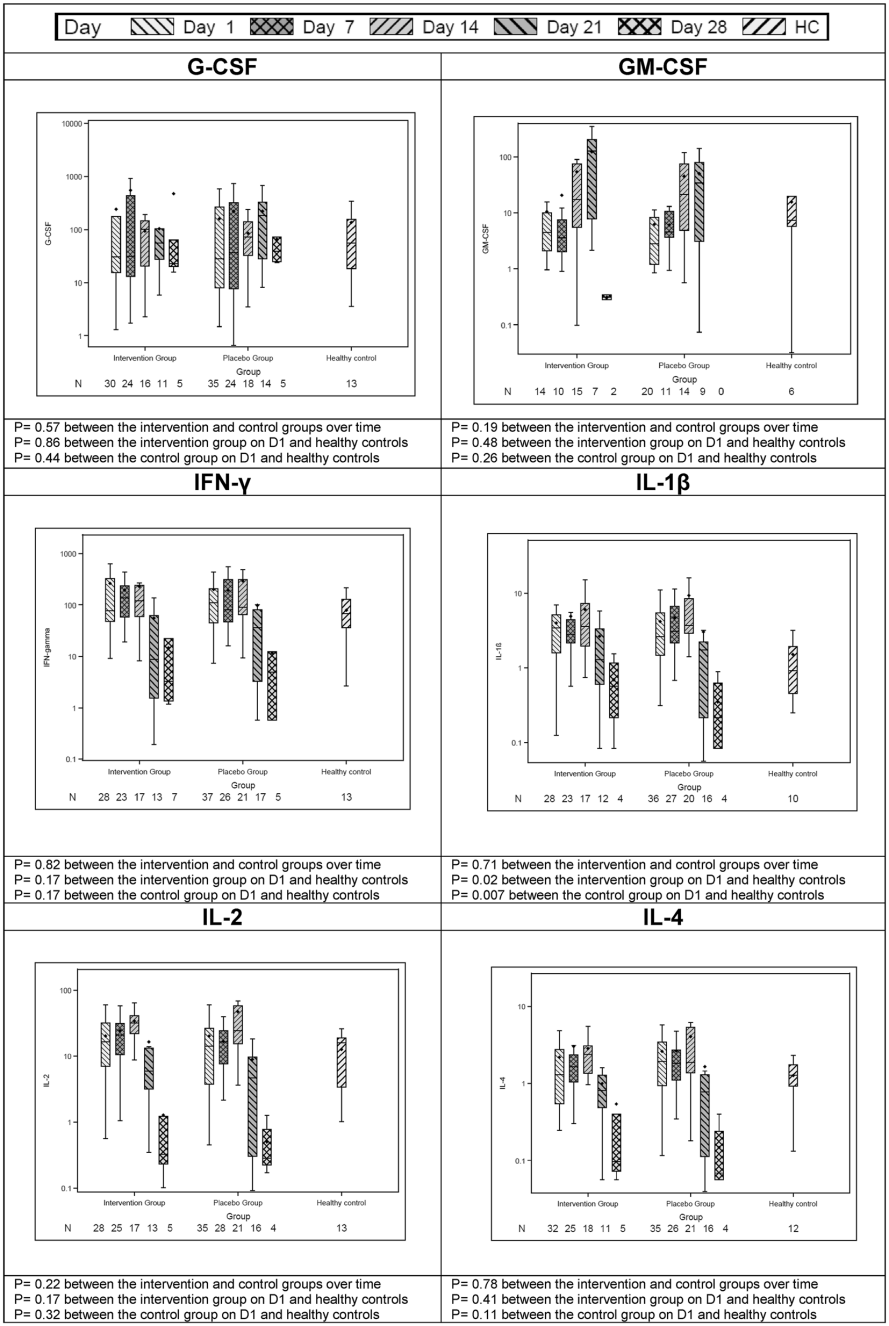
Serial measurements of selected plasma cytokine concentrations in patients treated with interferon-β1b and lopinavir–ritonavir, patients treated with placebo and healthy control. All cytokine levels were calculated based on mean fluorescent intensity and reported in pg/mL. We compared serial cytokine levels between patients treated with interferon-β1b and lopinavir–ritonavir and patients treated with placebo using a mixed linear model. We compared D1 values in both groups with those of healthy control using Mann–Whitney U test. G-CSF: granulocyte-colony stimulating factor; GM-CSF: granulocyte–macrophage colony-stimulating factor; IFN: interferon; IL: interleukin.

On day 1 (before the administration of study drugs), the following plasma cytokine concentrations were elevated in both groups compared to healthy subjects: interleukin (IL)-1β, IL-6, IL-8, IL-10, monocyte chemo-attractant protein (MCP)-1 and tumor necrosis factor (TNF)-α (Table 1 and Fig. 1).

### Effect of treatment by levels of cytokines and time to treatment

**Figure 2:**
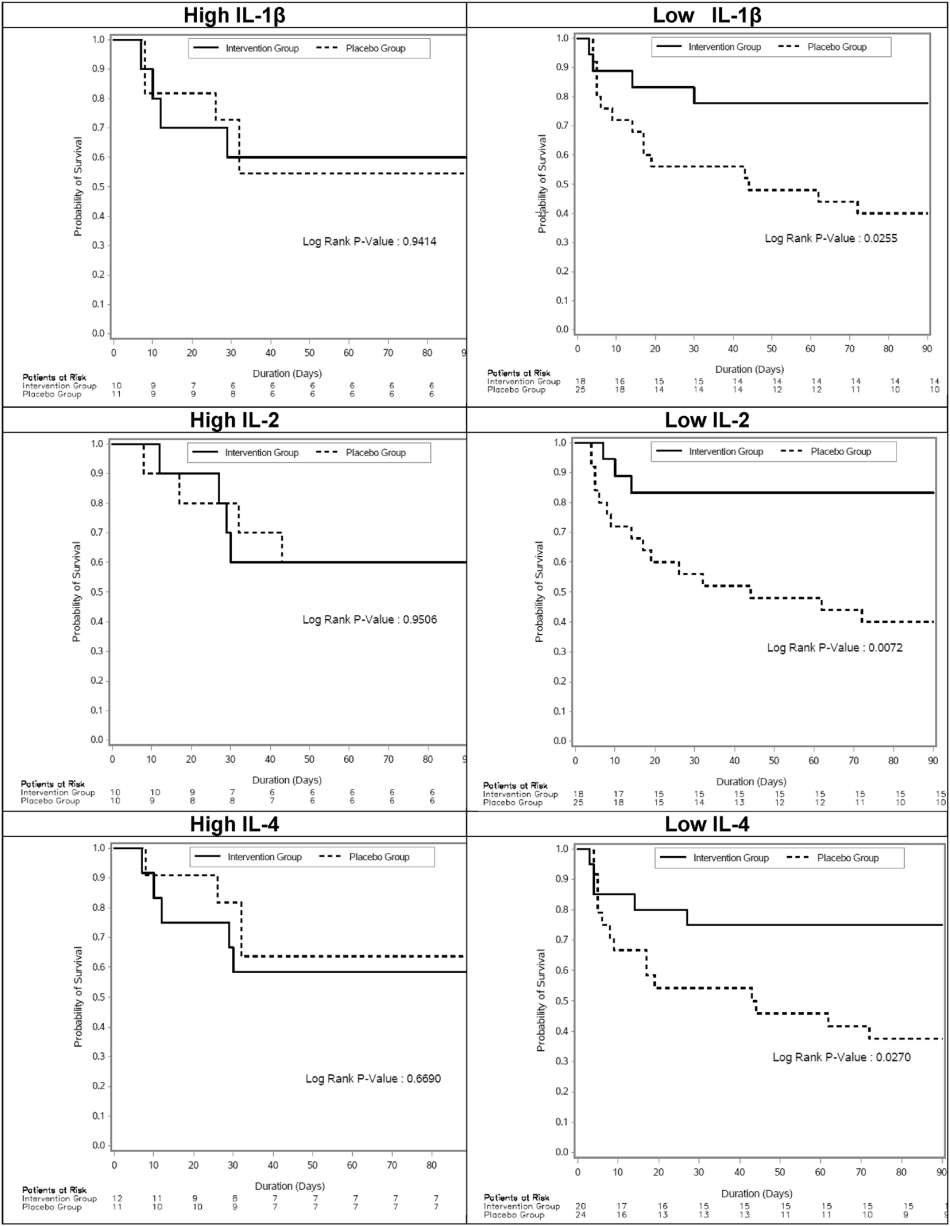
Kaplan–Meier time-to-event curves for mortality for patients with Middle East Respiratory Syndrome treated with interferon-β1b and lopinavir–ritonavir or placebo categorized into two subgroups of higher and lower levels of each of selected cytokines using the upper tertile (67%) as a cutoff point. Each cytokine is presented into two graphs based on the high (left panel) and low (right panel) levels at day 0. The number of patients at risk in treatment and placebo groups are presented. IL: interleukin.

**Figure 3:**
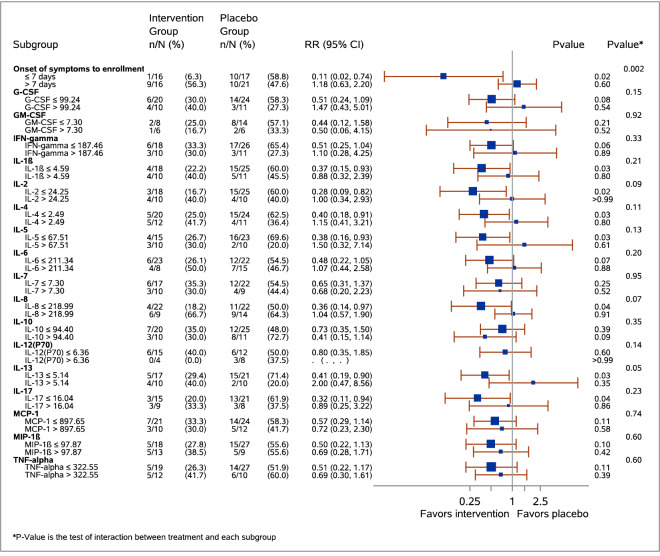
Forest plot demonstrating the association of interferon-β1b and lopinavir–ritonavir treatment on 90-day mortality in patients with Middle East respiratory syndrome categorized into two subgroups of early and late treatment and according to higher and lower levels of each of the cytokines using the upper tertile (67%) as a cutoff point. The results are displayed as relative risks and 95% confidence intervals (CI), and p-values. Additionally, p-values for the interactions are reported. Plasma cytokine concentrations are expressed in pg/ml. There was heterogeneity of treatment effect on 90-day-mortality according to the level of IL-2, IL-8 and IL-13 as demonstrated by testing for interaction (p-value for interaction = 0.09, 0.07, and 0.05, respectively) while the interaction was were not significant for other cytokines (p > 0.1). G-CSF: granulocyte-colony stimulating factor; GM-CSF: granulocyte–macrophage colony-stimulating factor; IFN: interferon; IL: interleukin; MCP: Monocyte chemo-attractant protein; MIP: Macrophage inflammatory protein; TNF: tumor necrosis factor.

Treatment with recombinant interferon-β1b and lopinavir–ritonavir generally appeared to reduce 90-day mortality in patients with lower cytokine levels on study day 1 (IL-1β, IL-2, IL-4, IL-5, IL-8, IL-13, IL-17, p values < 0.05) but not those with higher cytokine levels as demonstrated on Kaplan–Meier curves (Fig. 2). There was heterogeneity of treatment effect on 90-day-mortality according to the level of IL-2, IL-8 and IL-13 as demonstrated by testing for interaction (p-value for interaction = 0.09, 0.07 and 0.05, respectively) while interactions were not significant for other cytokines (p > 0.1) (Fig. 3).

### Effect of time between symptom onset and randomization

The distribution of the time of “onset of symptoms to randomization” and the cumulative number of deaths is shown in Figure S1. Among patients who were randomized within 5 days of symptom onset, there were no deaths 0/12 (0.0%) in the intervention group as compared to 8/11 (72.7%) in the placebo group. Similar to the published primary analysis of the MIRACLE trial^9^, early but not late treatment was effective in this subset of patients with MERS (p-value for interaction 0.002, Fig. 2).

### Cytokine changes over time

There were no differences in the plasma cytokine levels between the intervention and control groups over time (Fig. 1). Granulocyte–macrophage colony-stimulating factor (GM-CSF), IL-13 and IL-17 were higher over time in patients with onset of symptoms ≤ 7 days compared to > 7 days (Figure S2). Interferon-γ, IL-1β, IL-8, IL-17 and MCP-1 were higher over time in patients who did not survive compared to those who survived (Figure S3).

### Exploratory analyses

The results of exploratory analyses defining the higher and lower levels of each of the cytokines by using the median or the lower tertile (33%) as cutoff points showed statistically significant heterogeneity of treatment effect of recombinant interferon-β1b and lopinavir–ritonavir appeared on 90-day mortality according to IL-2 levels but not according other cytokines (p value <0.1) (Figure S4, Panel A and Panel B).

## Discussion

In this sub-study of a randomized clinical trial of hospitalized patients with MERS, we demonstrated that treatment with interferon-β1b and lopinavir–ritonavir treatment was associated with lower 90-day mortality among patients with lower, but not higher, cytokine levels at trial enrollment (specifically IL-2, IL-8, and IL-13), and among patients who were treated early.

We found the following cytokines to be elevated among hospitalized patients with MERS compared to healthy subjects: IL-1β, IL-6, IL-8, IL-10, MCP-1 and TNF-α. Increased proinflammatory cytokines have also been observed in our previous study, which showed elevations in plasma IL-3, IL-4, IL-6, IL-8, IL-17A, eotaxin and epidermal growth factor (EGF) compared to healthy controls^4^. Other studies of patients with MERS have also demonstrated elevated IL-1β, IL-1ra, IL-6, IL-8, IL-15, IL-17A, IP-10, TNF-α and interferon-γ^10–14^. Our study demonstrated that interferon-γ, IL-1β, IL-8, IL-17 and MCP-1 were higher over time in patients who did not survive compared to those who survived. Immune modulation therapy targeting pro-inflammatory cytokines requires further study in MERS.

The production of type I interferons (interferon-α and interferon-β) constitutes an early line of defense against multiple viral infections. Interferons mediate antiviral effects by inhibiting viral replication and modulating the host immune response^15^. Consequently, type I interferons have been used commonly in the treatment of MERS, but prior observational studies demonstrated inconsistent results^16–19^. The largest cohort of critically ill patients with MERS (n = 349), showed that ribavirin and recombinant interferon (α2a, α2b or β1a) therapy was not associated with a reduction in 90-day mortality or faster MERS-coronavirus RNA clearance, although treatment was generally late in this cohort (median time from onset of symptoms to treatment [9.0 days (6.0, 12.0)]^20^. However, results from the MIRACLE trial demonstrated that treatment with interferon-β1b and lopinavir–ritonavir resulted in a reduction in mortality and that the effect occurred mainly if treatment was started early (within 7 days of symptom onset). The benefit of early interferon therapy has been demonstrated in a murine model, in which early therapy initiation (one day after viral inoculation infection) protected mice from lethal MERS-CoV infection by inhibiting viral replication and inflammatory cytokine production^5^. On the other hand, delayed interferon-β administration in the same model caused remarkable increases in inflammatory cytokine levels and lethal disease^5^. Our human data indicate that the survival of MERS patients treated with interferon-β1b and lopinavir–ritonavir is influenced by both timing and the baseline inflammatory status. Unlike the two animal model studies in MERS and SARS, we did not observe a harm signal with late therapy with interferon-β1b and lopinavir–ritonavir^5,6^.

Heterogeneity of treatment effect based on the underlying inflammatory status has been observed in patients with ARDS. Post hoc data from several randomized controlled trials have demonstrated that approximately 30% of ARDS patients could be categorized as having a relative hyperinflammatory sub-phenotype and 70% of patients as having a relative hypoinflammatory sub-phenotype^21–24^. The two sub-phenotypes of ARDS appear to differ in response to certain therapies, including simvastatin, fluid management and positive end-expiratory pressure (PEEP)^23^. The heterogeneity of treatment effect in the current study appears to be more obvious with the timing of therapy than with cytokines. This may be related to the well-known variation in cytokine levels among patients.

The findings of this study should be considered in light of its strengths and weaknesses, including the post hoc nature of the analysis. This is the first study to assess the heterogeneity of interferon-β1b and lopinavir–ritonavir treatment effect by plasma cytokine levels and time from MERS symptom onset. Additionally, data were derived and analyzed from a multicenter, double-blind, randomized trial. The number of patients who were included in the study, albeit small, is considerable for a rare disease such as MERS. Nevertheless, the sample size likely reduced the study's power to detect modest differences and did not permit categorization of inflammatory status based on cytokine profile, using, for example, latent class analysis. For that reason, we categorized patients based on upper tertile versus lower two tertiles, an approach that is supported by other studies on MERS and ARDS^4,21–24^. Exploratory analyses using other cutoff points confirmed that this approach of using the upper tertile provided the best differentiation of those who may or may not respond to treatment with interferon-β1b and lopinavir–ritonavir compared to the median or lower tertile cutoffs.

In conclusion, treatment of hospitalized MERS patients with interferon-β1b and lopinavir–ritonavir treatment was associated with lower 90-day mortality among patients with lower but not higher IL-2, IL-8, and IL-13 levels, and among patients who were treated early in their illness course. The findings of the study could serve as the basis for future studies with larger sample sizes to evaluate whether the assessment of inflammatory status can help in identifying patients with MERS who may benefit from interferon-β1b and lopinavir–ritonavir or other emerging therapeutics for MERS.

## Methods

### Study design

This sub-study was a post hoc analysis for the MIRACLE trial (ClinicalTrials.gov number, NCT02845843)^7–9^. In this trial, 95 patients were randomly assigned to receive recombinant interferon β-1b and lopinavir–ritonavir (intervention) or placebo for 14 days. The study found that combined treatment resulted in lower 90-day mortality in hospitalized patients with laboratory-confirmed MERS. The study was sponsored by King Abdullah International Medical Research Center, Riyadh, Saudi Arabia. A detailed description of the study has already been published^7–9^.

### Blood samples and cytokine assay

For this sub-study, blood samples were collected from enrolled patients from the three main recruiting sites in Riyadh, Saudi Arabia (n=70), between November 2016 through April 2020. Patients enrolled from other cities were not included in this sub-study because of logistic reasons related to the shipping and handling of samples. Blood samples were collected in heparin EDTA tubes on days 1, 7, 14, 21 and 28 of enrollment, where the blood sample on day 1 (the day of enrollment) was obtained before the administration of study drugs. Samples were also collected from 10 healthy individuals to serve as controls. Samples were centrifuged for 10 min at 1000×*g* at 4 °C, and plasma was isolated and stored in cryo-tubes at − 80 °C until the day of analysis. A panel of 17 cytokines was measured in duplicates using Milliplex panel (Bio-Plex Pro Human Cytokine Grp I Panel 17-Plex, BIO-RAD, Cat:#M5000031YV, USA), according to the manufacturer's instructions. The cytokine panel included granulocyte-colony stimulating factor (G-CSF), GM-CSF, interferon-γ, IL-1β, IL-2, IL-4, IL-5, IL-6, IL-7, IL-8, IL-10, IL-12 (P70), IL-13, IL-17, MCP-1, macrophage inflammatory protein (MIP)-1β and TNF-α. All cytokine levels were calculated based on mean fluorescent intensity using Luminex FLEXMAP 3D instrument system and xPONENT software v4.2 (Luminex Corporation, Austin, USA) and reported in pg/mL using a five-parameter logistical regression of the standard curve as a fitting method by using the Belysa software v1.1.0 (Merck KGaA, Darmstadt, Germany).

### Clinical data

We collected baseline data on demographics, comorbidities, the severity of the disease, laboratory parameters and organ support at baseline among study patients in the two groups, study interventions and co-interventions during hospitalization. The primary outcome was 90-day all-cause mortality. Secondary outcomes included 28-day mortality, ICU and hospital mortality, organ support-free days calculated at 90 days, subjects who died were assigned 0 free days (including free days of supplemental oxygen, invasive or non-invasive mechanical ventilation, renal replacement therapy, vasopressor therapy, extracorporeal membrane oxygenation), ICU-free days, time to MERS-CoV RNA clearance among all patients and survivors.

### Statistics

Categorical variables were represented as frequency and percentage (%), and continuous variables as medians and interquartile ranges (Q1, Q3). For categorical variables, the Chi-square test or Fisher’s exact test was used, and for continuous variables, Student’s t-test or the Mann–Whitney U test was used as appropriate.

We compared serial cytokine levels between patients treated with interferon-β1b and lopinavir–ritonavir and patients treated with placebo using a mixed linear model. We compared enrollment (D1) values in both groups with those of healthy control using Mann–Whitney U test. We categorized patients into two subgroups of higher and lower levels of each of cytokines using the upper tertile (67%) as a cutoff point, an approach that is supported by other studies on MERS and ARDS that showed that approximately one-third of patients fall within the relative hyperinflammatory sub-phenotype^4,21–24^. We performed another exploratory analysis defining the higher and lower levels of each cytokine using the median or the lower tertile (33%) as cutoff points. We expressed the treatment effect by reporting absolute and relative risk reduction and 95% confidence interval. Log binomial regression was used and tested for heterogeneity of treatment effect between the two subgroups by testing for interaction. We conducted survival analysis and reported Kaplan–Meier survival curves and the results of the log-rank test. We also compared serial cytokine levels among patients treated within ≤ 7 days, patients treated after 7 days of symptom onset and healthy controls. Similar analyses were performed for serial cytokine levels among survivors, non-survivors and healthy control. All analyses were performed using SAS 9.4 (SAS Institute, Cary, NC). We did not test for multiplicity, given the exploratory nature of this analysis. Statistical tests for variables were performed using a two-sided alpha value of 0.05 to denote the significance level. p-values < 0.1 for interaction were considered significant, given the exploratory nature of the analysis.

### Study approval

The Institutional Board Review of the Ministry of National Guard Health Affairs (RC 15/142/R), The Research Ethic Committee of the Prince Sultan Military Medical City (Project No: 868), and the Prince Mohammad Bin Abdulaziz Hospital (16-406E1, Institutional Review Board of King Fahad Medical City—Ministry of Health) reviewed the study and approved it in accordance with the ethical standards of the responsible committees on human experimentation and with the Declaration of Helsinki. Informed consent was obtained for participation in the main study and this current study.

## Supplementary Information

Supplementary Information.

## Data Availability

The datasets generated and/or analyzed during the current study are available from the corresponding author upon reasonable request once all planned analyses have been completed and published or presented and after signing sharing agreement in accordance with the policies of KAIMRC.

## References

[1] Arabi YM (2017). Middle East Respiratory Syndrome. N. Engl. J. Med..

[2] Hemida MG, Ali AM, Alnaeem A (2021). The Middle East respiratory syndrome coronavirus (MERS-CoV) nucleic acids detected in the saliva and conjunctiva of some naturally infected dromedary camels in Saudi Arabia -2019. Zoonoses Public Health.

[3] Aljasim TA (2020). High rate of circulating MERS-CoV in dromedary camels at slaughterhouses in Riyadh, 2019. Viruses.

[4] Arabi YM (2021). Inflammatory response and phenotyping in severe acute respiratory infection from the Middle East respiratory syndrome coronavirus and other etiologies. Crit. Care Med..

[5] Channappanavar R (2019). IFN-I response timing relative to virus replication determines MERS coronavirus infection outcomes. J. Clin. Invest..

[6] Channappanavar R (2016). Dysregulated type I interferon and inflammatory monocyte-macrophage responses cause lethal pneumonia in SARS-CoV-infected mice. Cell Host Microbe.

[7] Arabi YM (2020). Treatment of Middle East respiratory syndrome with a combination of lopinavir/ritonavir and interferon-β1b (MIRACLE trial): Statistical analysis plan for a recursive two-stage group sequential randomized controlled trial. Trials.

[8] Arabi YM (2018). Treatment of Middle East Respiratory Syndrome with a combination of lopinavir-ritonavir and interferon-β1b (MIRACLE trial): Study protocol for a randomized controlled trial. Trials.

[9] Arabi YM (2020). Interferon Beta-1b and lopinavir-ritonavir for Middle East respiratory syndrome. N. Engl. J. Med..

[10] Kim ES (2016). Clinical progression and cytokine profiles of Middle East respiratory syndrome coronavirus infection. J. Korean Med. Sci..

[11] Shin HS (2019). Immune responses to Middle East respiratory syndrome coronavirus during the acute and convalescent phases of human infection. Clin. Infect. Dis..

[12] Mahallawi WH, Khabour OF, Zhang Q, Makhdoum HM, Suliman BA (2018). MERS-CoV infection in humans is associated with a pro-inflammatory Th1 and Th17 cytokine profile. Cytokine.

[13] Lau SKP (2013). Delayed induction of proinflammatory cytokines and suppression of innate antiviral response by the novel Middle East respiratory syndrome coronavirus: Implications for pathogenesis and treatment. J. Gen. Virol..

[14] Faure E (2014). Distinct immune response in two MERS-CoV-infected patients: Can we go from bench to bedside?. PLoS ONE.

[15] Totura AL, Baric RS (2012). SARS coronavirus pathogenesis: Host innate immune responses and viral antagonism of interferon. Curr. Opin. Virol..

[16] Khalid M (2015). Ribavirin and interferon-alpha2b as primary and preventive treatment for Middle East respiratory syndrome coronavirus: A preliminary report of two cases. Antivir. Ther..

[17] Al-Tawfiq JA, Momattin H, Dib J, Memish ZA (2014). Ribavirin and interferon therapy in patients infected with the Middle East respiratory syndrome coronavirus: An observational study. Int.J. Infect. Dis..

[18] Shalhoub S (2015). IFN-alpha2a or IFN-beta1a in combination with ribavirin to treat Middle East respiratory syndrome coronavirus pneumonia: A retrospective study. J. Antimicrob. Chemother..

[19] Omrani AS (2014). Ribavirin and interferon alfa-2a for severe Middle East respiratory syndrome coronavirus infection: A retrospective cohort study. Lancet. Infect. Dis.

[20] Arabi YM (2019). Ribavirin and interferon therapy for critically ill patients with Middle East respiratory syndrome: A multicenter observational study. Clin. Infect. Dis..

[21] Calfee CS (2014). Subphenotypes in acute respiratory distress syndrome: Latent class analysis of data from two randomised controlled trials. Lancet Respir. Med..

[22] Famous KR (2017). Acute respiratory distress syndrome subphenotypes respond differently to randomized fluid management strategy. Am. J. Respir. Crit. Care Med..

[23] Calfee CS (2018). Acute respiratory distress syndrome subphenotypes and differential response to simvastatin: Secondary analysis of a randomised controlled trial. Lancet Respir. Med..

[24] Sinha P (2018). Latent class analysis of ARDS subphenotypes: A secondary analysis of the statins for acutely injured lungs from sepsis (SAILS) study. Intensive Care Med..

