# Supplementary material for: Heterogeneity of treatment effect of interferon-β1b and lopinavir–ritonavir in patients with Middle East respiratory syndrome by cytokine levels
Source: Sci Rep. 2022 Oct 28;12:18186. doi: 10.1038/s41598-022-22742-8 (PMC9616407; doi:10.1038/s41598-022-22742-8)
Supplement: Supplementary file 1 — Supplementary Information. [file 41598_2022_22742_MOESM1_ESM.docx]

**Supplementary file**

**Heterogeneity of Treatment Effect of Interferon-β1b and Lopinavir–ritonavir in Patients with Middle East Respiratory Syndrome by Cytokine Levels**

Yaseen M. **Arabi**,^1^ Ayed Y **Asiri**,^2^ Abdullah M **Assiri**,^3^ Mashan L. Abdullah, ^4^ Haya A Aljami, ^5^ Hanan H **Balkhy**,^6^ Majed **Al Jeraisy**,^7^ Yasser **Mandourah**,^8^ Sameera **AlJohani**,^9^ Shmeylan **Al Harbi**,^10^ Hani A. Aziz **Jokhdar**,^11^ Ahmad M. **Deeb**,^12^ Ziad A. **Memish**,^13^ Jesna **Jose**,^14^ Sameeh **Ghazal**,^15^ Sarah Al **Faraj**,^16^ Ghaleb A. **Al Mekhlafi**,^17^ Nisreen Murad **Sherbeeni**,^18^ Fatehi Elnour **Elzein**,^19^ Frederick G. **Hayden**,^20^ Robert A. **Fowler**,^21^ Badriah M **AlMutairi**,^22^ Abdulaziz **Al-Dawood**,^23^ Naif Khalaf **Alharbi**,^24^

**Contents**

**MIRACLE management and writing committees, Data Monitoring Committee and MIRACLE site collaborators**

**Supplementary tables**

**Table S1:** Study interventions and co-interventions.

**Supplementary figures**

**Figure S1:** Cumulative number of deaths according to the time from onset of symptoms to therapy with interferon-β1b and lopinavir-ritonavir or placebo.

**Figure S2:** Serial measurements for cytokines in patients treated within ≤7 days, patients treated after 7 days of symptom onset and healthy control.

**Figure S3:** Serial measurements for cytokines in survivors, non-survivors and healthy control.

**Figure S4:** Exploratory analyses for defining the higher and lower levels of each of cytokines using the median or the median or lower tertile (33%) as cutoff points.

| **MIRACLE management and writing committees, Data Safety Monitoring Board and MIRACLE site collaborators** | |
| --- | --- |
| Management Committee | Yaseen M. **Arabi**  Adel **Alothman**  Hanan H **Balkhy**  Abdulaziz **Al-Dawood**  Sameera **AlJohani**  Shmeylan **Al Harbi**  Majed **Al Jeraisy**  Ahmad M. **Deeb**  Naif Khalaf **Alharbi**  Badriah M **AlMutairi**  Jesna **Jose**  Mohamed A. **Hussein**  Mohammed **Al Muhaidib** |
| Writing Committee | Yaseen M. **Arabi**  Jesna **Jose**  Mohamed A. **Hussein**  Ahmad M. **Deeb**  Robert A. **Fowler**  Frederick G. **Hayden** |
| Data Safety Monitoring Board | Greg S **Martin**  David A **Schoenfeld**  Sharon L **Walmsley**  Shannon **Carson** |
| **Collaborators - the Saudi Critical Care Trials Group** |  |
| King Saud bin Abdulaziz University for Health Sciences and King Abdullah International Medical Research Center, Riyadh, Saudi Arabia | Yaseen M. **Arabi**  Adel **Alothman**  Hanan H **Balkhy**  Abdulaziz **Al-Dawood**  Sameera **AlJohani**  Shmeylan **Al Harbi**  Suleiman **Kojan**  Majed **Al Jeraisy**  Naif Khalaf **Alharbi**  Ahmad M. **Deeb**  Badriah M **AlMutairi**  Jesna **Jose**  Mohamed A. **Hussein**  Mohammed **Al Mohaidib** Musharaf **Sadat**  Hala **Al Anizi**  Reggie **Dael**  Mohamed W. **Alenazi**  Haya A. **Aljami**  Ali **Alshehri**  Ramesh K **Vishwakarma** |
| Ministry of Health, Saudi Arabia | Abdullah M **Assiri**  Hani A. Aziz **Jokhdar**  Mohammad **AlMazroa**  Fawaz **Al-Rasheedi** |
| Prince Mohammed bin Abdulaziz Hospital, Riyadh, Saudi Arabia | Ayed Y. **Asiri**  Ziad A **Memish**  Sameeh S **Ghazal**  Sarah H **Alfaraj**  Mohammed **Alshaikh**  Dhaifallah Saud **Alotaibi**  Mostafa **Rajab**  Fatima Emieraiza P. **Isdung**  Chloe D. **Abinal**  Ruchil S. **Escobanez**  Carlos B. **landingin**  Samah **Badamas**  Norah Abdullatif **Hawsawi**  Hanan **Alanazi**  Anwar Ali **Mohammed**  Hail **Al Nono**  Ali Othman **Alkahlaf**  Fahad **Al Daeaji**  Ahmed **Madi**  Abdulrahaman **Idrees** |
| King Fahad Medical City, Riyadh, Saudi Arabia | Abdullah **Al Motairi**  Mushira **Al Enani**  Alaa **Alqurashi**  Fatimah **Alenezi**  Nada **Alkhani** |
| Prince Sultan Military Medical City, Riyadh, Saudi Arabia | Yasser **Mandourah**  Ghaleb A. **AlMekhlafi**  Nisreen Murad **Sherbeeni**  Fatehi Elnour **Elzein**  Shatha Anwar **Al Samarrai**  Rima E **Mahamed**  Abdulrauf Ahmed **Malibary**  Bander **Al Anezi**  Ma. Raylin **Cubio**  Melvin **Salunga**  Shatha Moayad **Awad**  Maha E. **Aljuhani**  Ghena **Jaber**  Adnan **Alghamedi**  Osama **Elfaki**  Najlaa **Almutairi** |
| King Saud Medical City, Riyadh, Saudi Arabia | Abdulrahman **AlHarthy**  Mohammed **AlSulaiman**  Ahmed **Mady**  Basheer **Abdulrhman** Tasmyia **Asaad** Gultakin **Bakirova**  Amany **Albraiky** Hamad **AlShahrany**  Huda **Mhawish**  Alva **Alcazar** |
| King Faisal Specialist Hospital & Research Center, Riyadh, Saudi Arabia | Khalid **Maghrabi** |
| King Abdulaziz Medical City, Jeddah, Saudi Arabia | Fahad **Al-Hameed**  Asim **Alsaedi**  Ohoud **Aloraabi**  Jalal **Refai**  Pansy **Elsamadisi**  Medhat S **Hendy**  Sara AbuBaker **Basher** |
| King Abdullah Medical Complex, Jeddah, Saudi Arabia | Mohamed Hatem A **Azzam**  Muhammed **Abduldhaher**  Wael **Bajhamoum**  Hala Ibrahim **Alnazawi**  Mohammad Nassar **Almadani**  Mohannad Saud **Alnefaie** |
| King Abdulaziz Hospital-Alahsa, Saudi Arabia | Jamal **Chalabi**  Yusri **Taha**  Javed **Memon**  Shahinaz **Bashir**  Ibraheem **Al-Dossary**  Saleh **Al Mekhloof** |
| King Fahad Hospital, Al-Madinah Al-Monawarah, Saudi Arabia | Ayman **Kharaba**  Ahmad **Al Jabri** |
| Ohoud Hospital, Al-Madinah Al-Monawarah, Saudi Arabia | Ayman **Kharaba**  Magdy **Farid**  Alawi **Alaidarous**  Wael **Alseraihi**  Husam **Shahada**  Jinish **Shimi** |
| Aseer Central Hospital, Abha, Saudi Arabia | Ali **Al Bshabshe**  Abdelmoniem **Al Bahar**  Wafa **Qadri**  Bensi **Mathew**  Ahmad Mushabab **Assiri**  Ali **Alhusin**  Nora **Assiri** |
| King Faisal Medical Complex, Taif, Saudi Arabia | Hanadi Mohamed Ahmed **Ouali**  Lamya **Al Zubaidi**  Rhea Mae **Gesulga**  Badr Ali **Al Harthy**  Abed Suryeh **Algothemi**  Rinu Mary Raju **Philip**  Rajani **Rajan** |
| Dammam Medical Complex, Dammam, Saudi Arabia | Shahab **Alsuliman**  Hajer **Aldossery**  Mohammed **Alnabi** |
| King Fahad Hospital, Hofuf, Saudi Arabi | Mahmoud **Albagshi** |
| King Khalid Hospital, Najran, Saudi Arabia | Abdulhadi Mohmmed **Bin Eshaq**  Abduelbagi D A **Altayb**  Ezaldeen H **Omer**  Salem Saleh **AlQirad**  Fatimah Awaadh **Balhareth**  Sutharani **Esakkimuthu**  Jubinamol **Chacko**  Maryjoy **Arquiza**  Dhanyamol C.**Babu** |
| University of Toronto, Sunnybrook Health Sciences Centre, Toronto, Canada | Robert A **Fowler** |
| University of Virginia School of Medicine, Charlottesville, VA, USA | Frederick G **Hayden** |

**Table S1:** Study interventions and co-interventions.

| **Variable** | **Intervention Group (N= 32)** | **Placebo Group**  **(N= 38)** | **P value** |
| --- | --- | --- | --- |
| **Study Interventions** |  |  |  |
| Time of onset of symptoms to randomization, day, median (IQR) | 7.5 (5.0, 11.0) | 8.0 (5.0, 12.0) | 0.52 |
| Time of admission to randomization, day, median (IQR) | 2.0 (1.0, 3.5) | 1.5 (1.0, 3.0) | 0.65 |
| Treatment duration, day, median (IQR) † | 14.0 (10.0, 14.0) | 14.0 (7.0, 14.0) | 0.69 |
| Number of lopinavir-ritonavir/placebo doses, median (IQR) | 25.0 (14.0, 28.0) | 26.0 (11.0, 28.0) | 0.91 |
| Number of interferon-β1b/placebo injection doses, median (IQR) | 7.0 (5.0, 7.0) | 7.0 (4.0, 7.0) | 0.95 |
| **Co-interventions** |  |  |  |
| Vasopressor therapy – no. (%) | 15 (46.9) | 25 (65.8) | 0.11 |
| Renal replacement therapy – no. (%) | 15 (46.9) | 20 (52.6) | 0.63 |
| Neuromuscular blockade – no. (%) | 14 (43.8) | 23 (60.5) | 0.16 |
| Invasive mechanical ventilation – no. (%) | 18 (56.3) | 29 (76.3) | 0.08 |
| Non-invasive mechanical ventilation – no. (%) | 3 (9.4) | 8 (21.1) | 0.18 |
| Extra corporeal membrane oxygenation (ECMO) – no. (%) | 2 (6.3) | 5 (13.2) | 0.44^^ |
| Corticosteroids – no. (%) | 19 (59.4) | 26 (68.4) | 0.43 |
| Duration -days, median (IQR) | 7.0 (5.0, 11.0) | 13.0 (6.0, 20.0) | 0.54 |

^†^Treatment duration: time from initiation of study drugs up to 14 days, death or hospital discharge whichever comes first.

**Figure S1:** Cumulative number of deaths according to the time from onset of symptoms to therapy with interferon-β1b and lopinavir-ritonavir (red) or placebo (blue).

**Figure S2:** Serial measurements for cytokines in patients treated within ≤7 days, patients treated after 7 days of symptom onset and healthy control. All cytokine levels were calculated based on mean fluorescent intensity and reported in pg/mL. We compared serial cytokine levels between patients treated within ≤7 days, patients treated after 7 days of symptom onset using a mixed linear model. We compared D1 values in both groups with those of healthy control using Mann–Whitney U test.

| 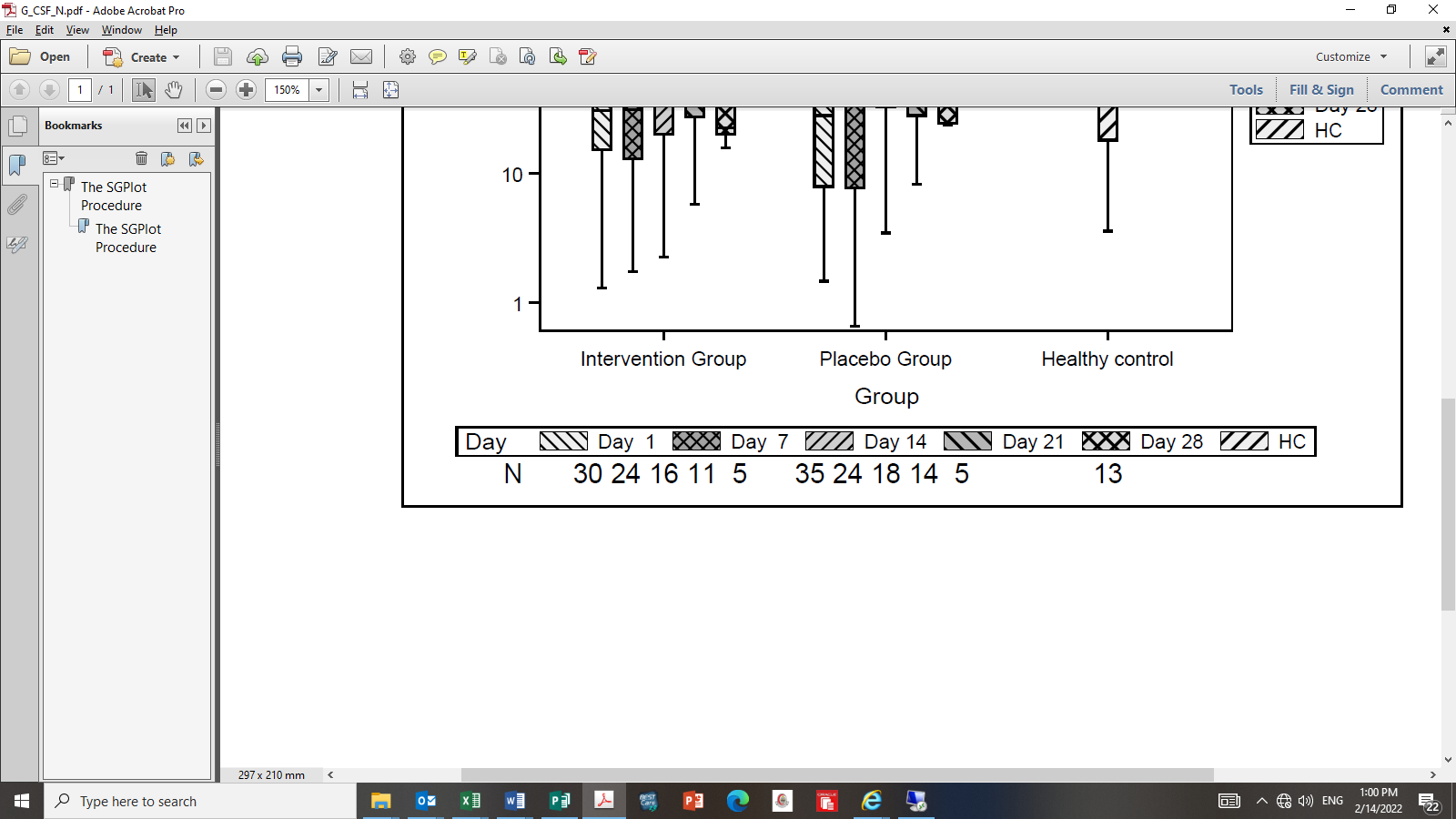 | |
| --- | --- |
| **G-CSF** | **GM-CSF** |
| 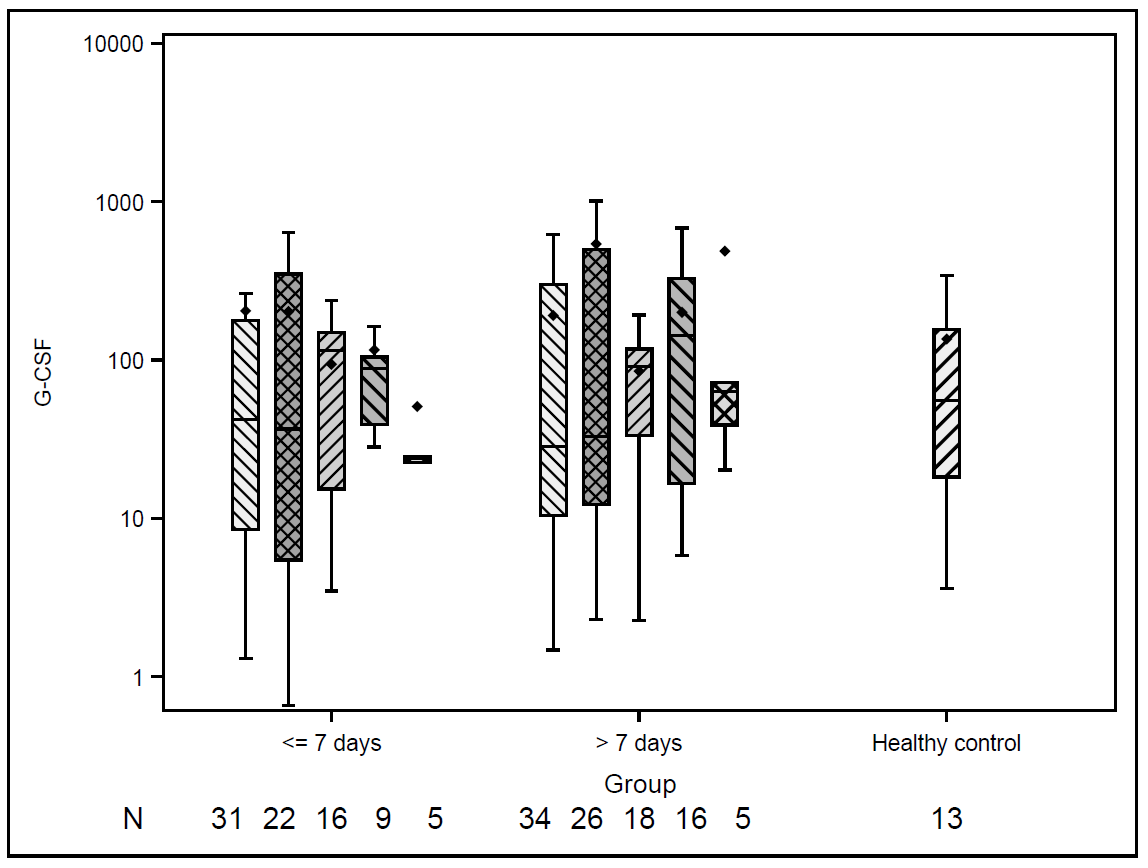 | 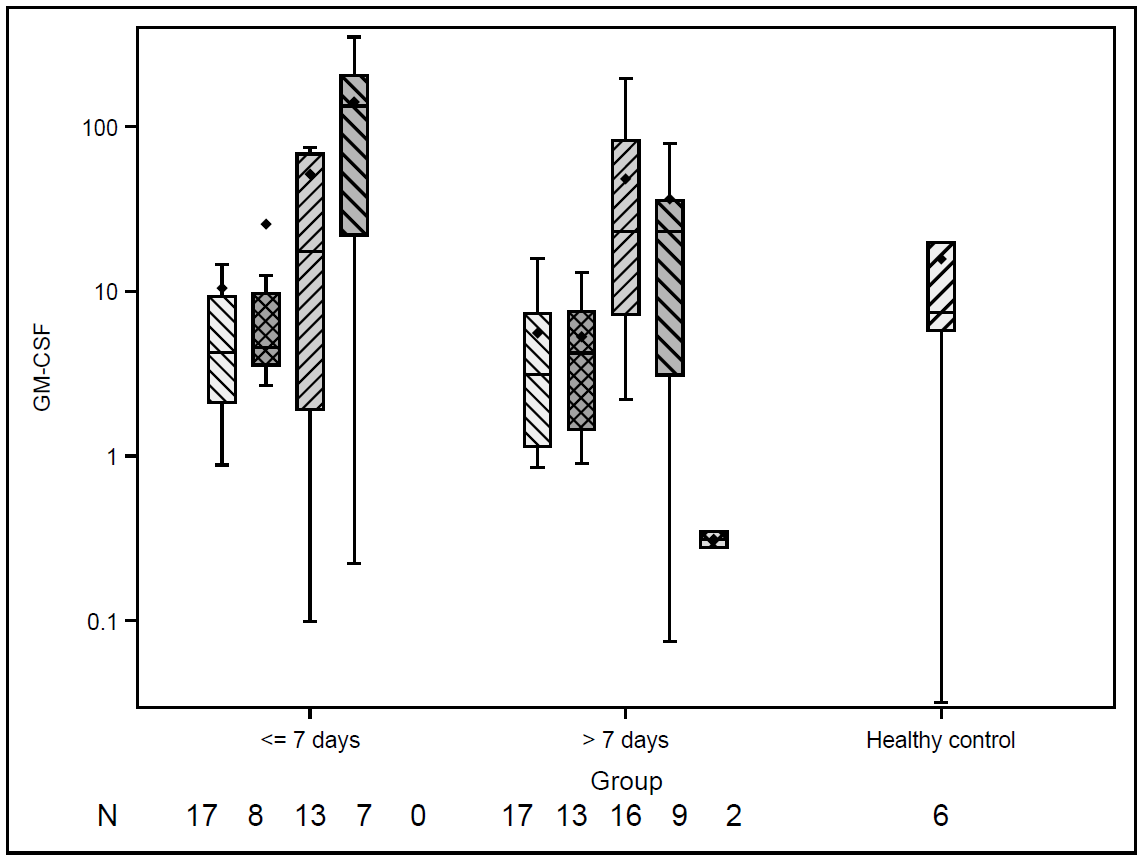 |
| P= 0.54 between the early and late treatment groups over time  P= 0.78 between the early treatment group on D1 and healthy controls  P= 0.50 between the late treatment group on D1 and healthy controls | P= 0.013 between the early and late treatment groups over time  P= 0.51 between the early treatment group on D1 and healthy controls  P= 0.22 between the late treatment group on D1 and healthy controls |
| **IFN-γ** | **IL-1β** |
| 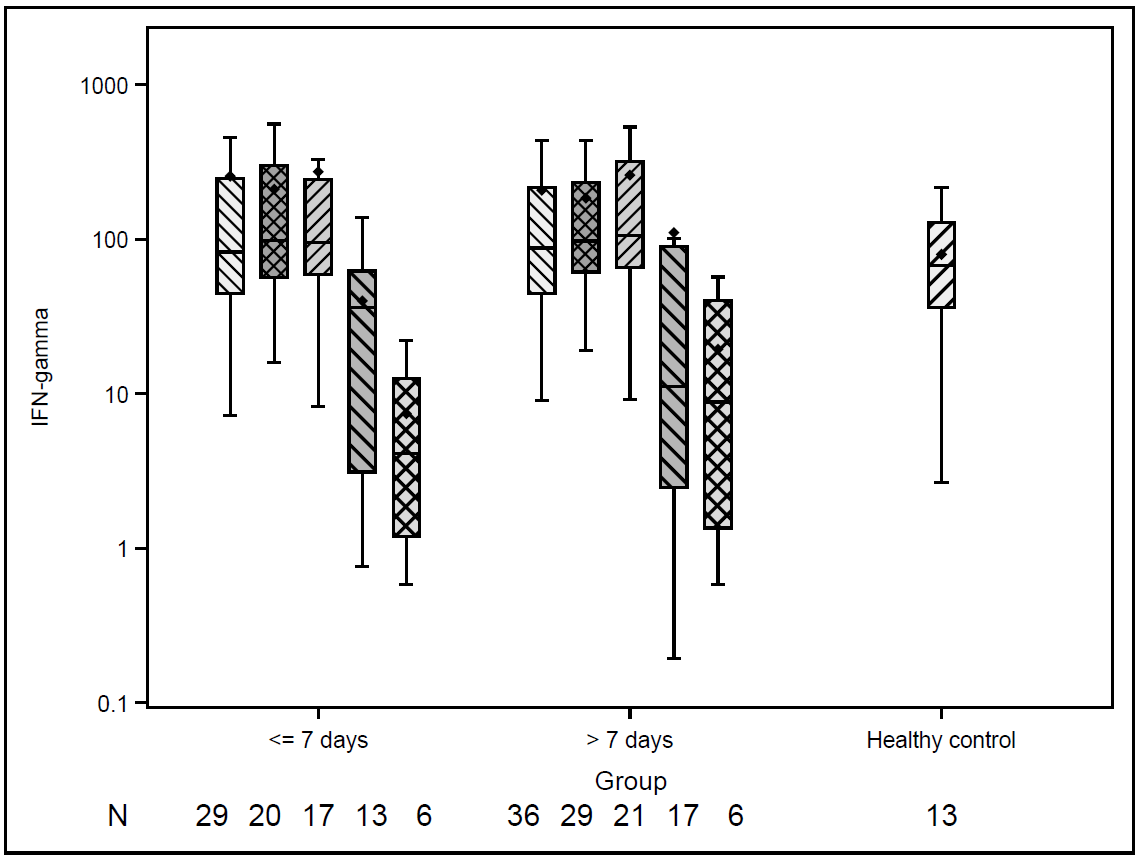 | 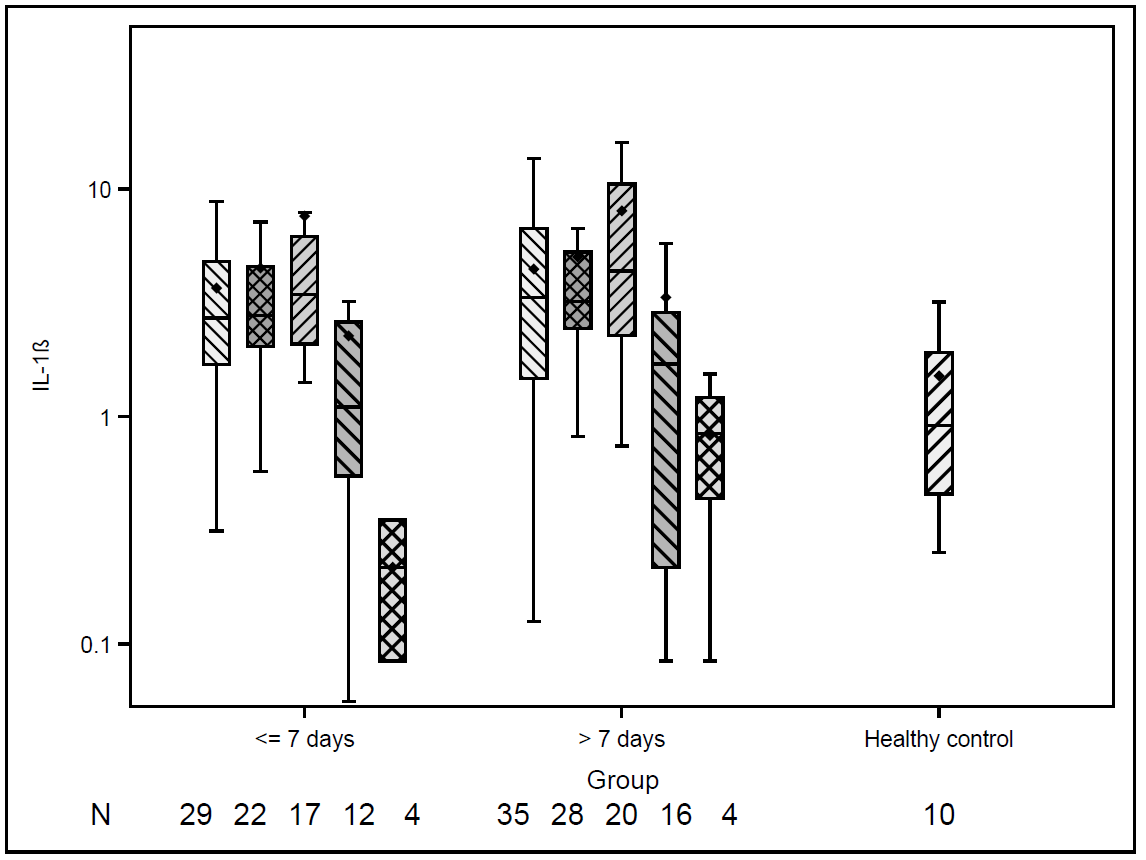 |
| P= 0.98 between the early and late treatment groups over time  P= 0.15 between the early treatment group on D1 and healthy controls  P= 0.19 between the late treatment group on D1 and healthy controls | P= 1.00 between the early and late treatment groups over time  P=0.0096 between early treatment group on D1 and healthy controls  P= 0.012 between the late treatment group on D1 and healthy controls |
| **IL-2** | **IL-4** |
| 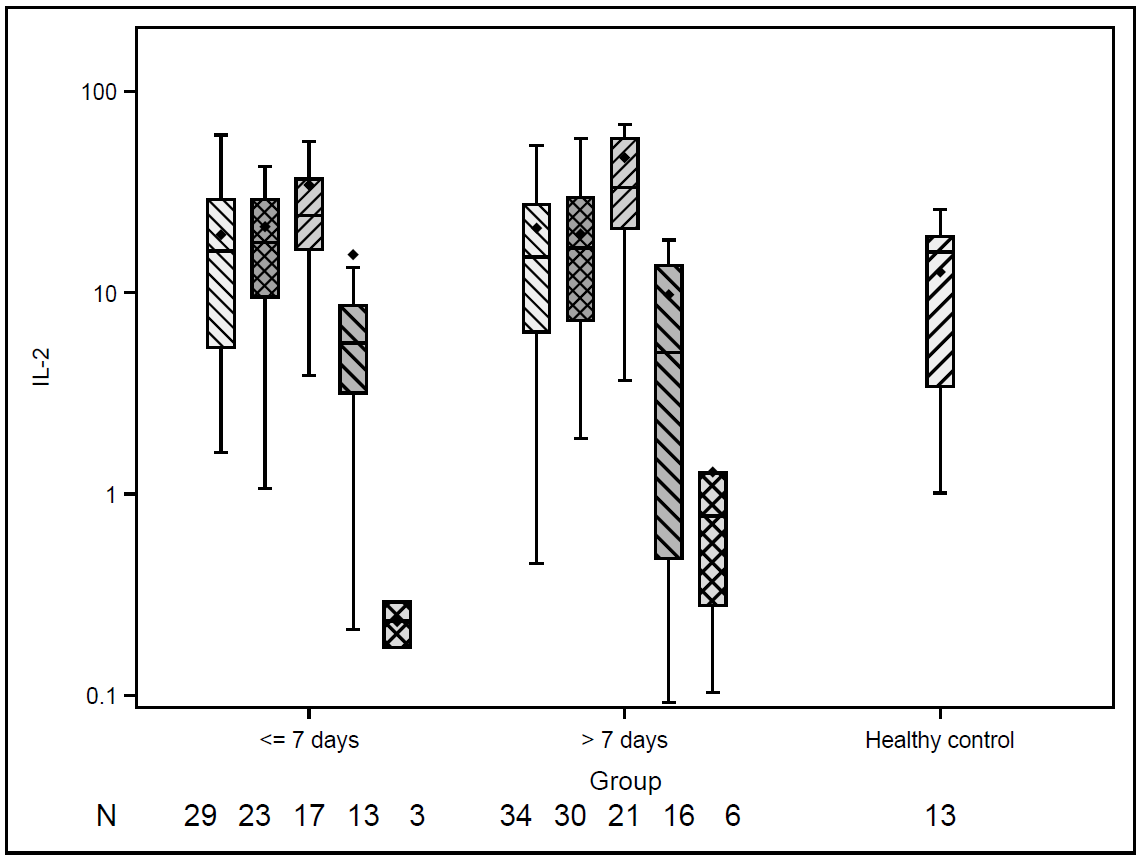 | 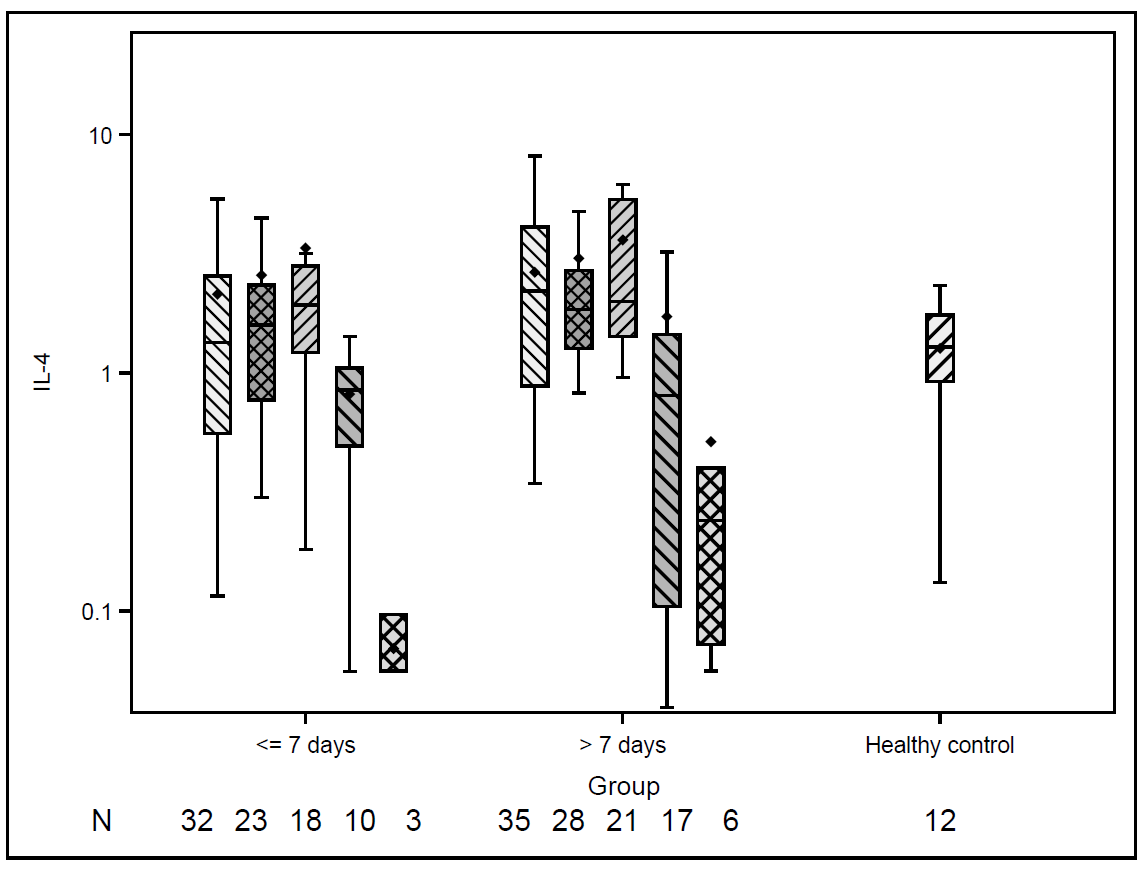 |
| P= 0.49 between the early and late treatment groups over time  P= 0.23between the early treatment group on D1 and healthy controls  P= 0.25 between the late treatment group on D1 and healthy controls | P= 1.00 between the early and late treatment groups over time  P= 0.44 between the early treatment group on D1 and healthy controls  P= 0.095 between the late treatment group on D1 and healthy controls |
| **IL-5** | **IL-6** |
| 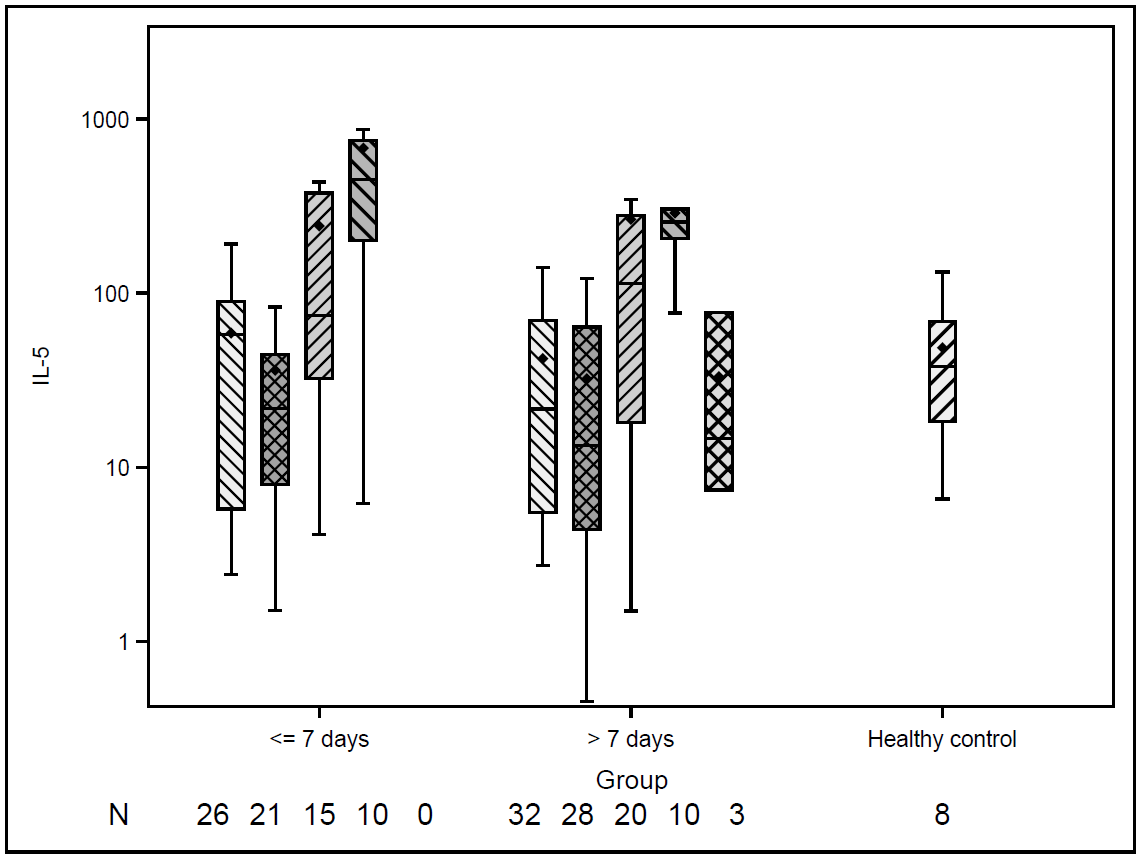 | 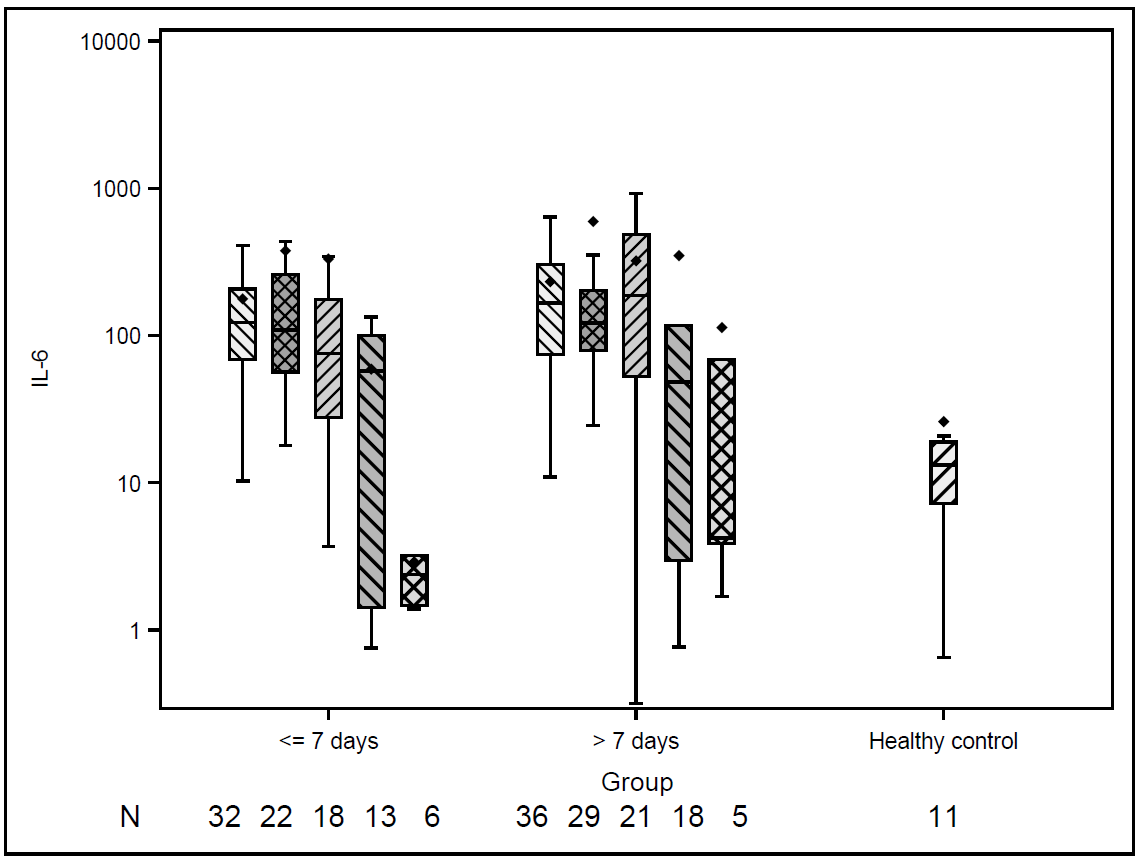 |
| P= 0.08 between the early and late treatment groups over time  P= 1.00 between the early treatment group on D1 and healthy controls  P= 0.43 between the late treatment group on D1 and healthy controls | P= 0.95 between the early and late treatment groups over time  P= <0.0001 between early treatment group on D1 and healthy controls  P= <0.0001 between late treatment group on D1 and healthy controls |
| **IL-7** | **IL-8** |
| 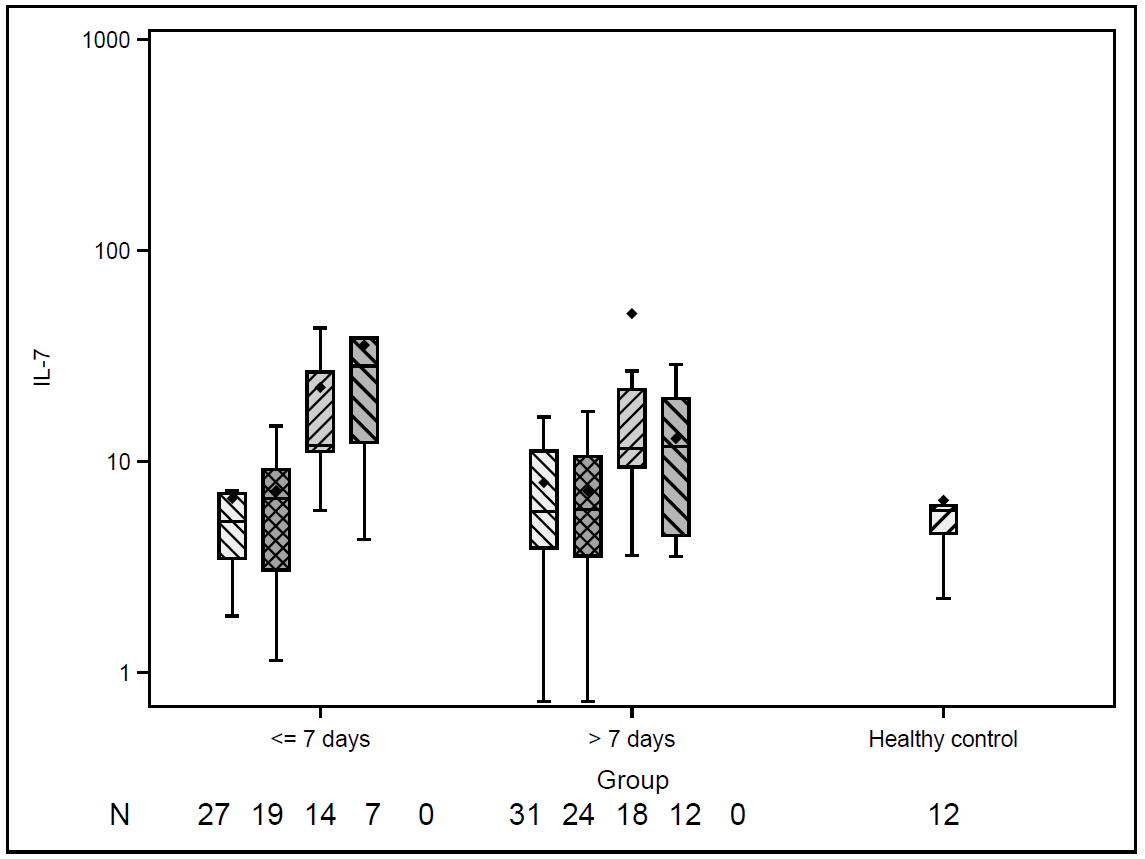 | 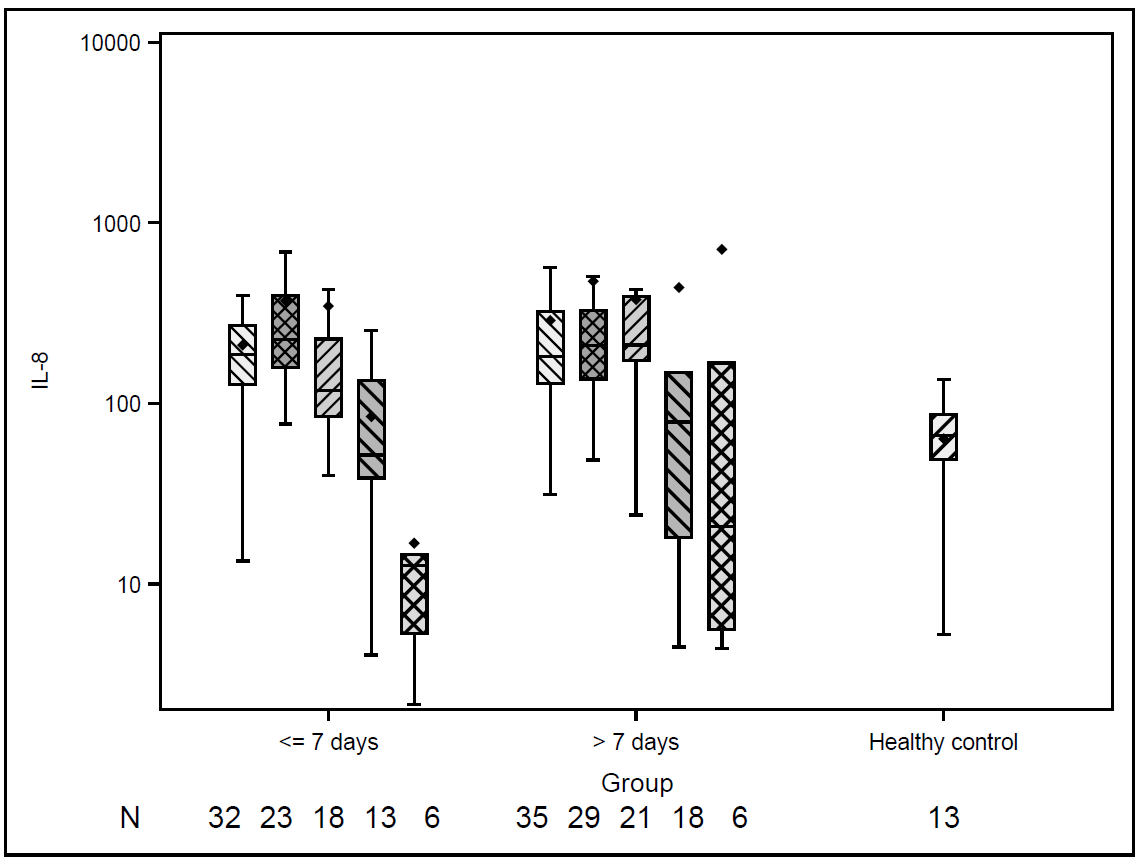 |
| P= 0.26 between the early and late treatment groups over time  P= 0.66 between the early treatment group on D1 and healthy controls  P= 0.87 between the late treatment group on D1 and healthy controls | P= 0.83 between the early and late treatment groups over time  P= 0.0002 between early treatment group on D1 and healthy controls  P= <0.0001 between late treatment group on D1 and healthy controls |
| **IL-10** | **IL-12 (P70)** |
| 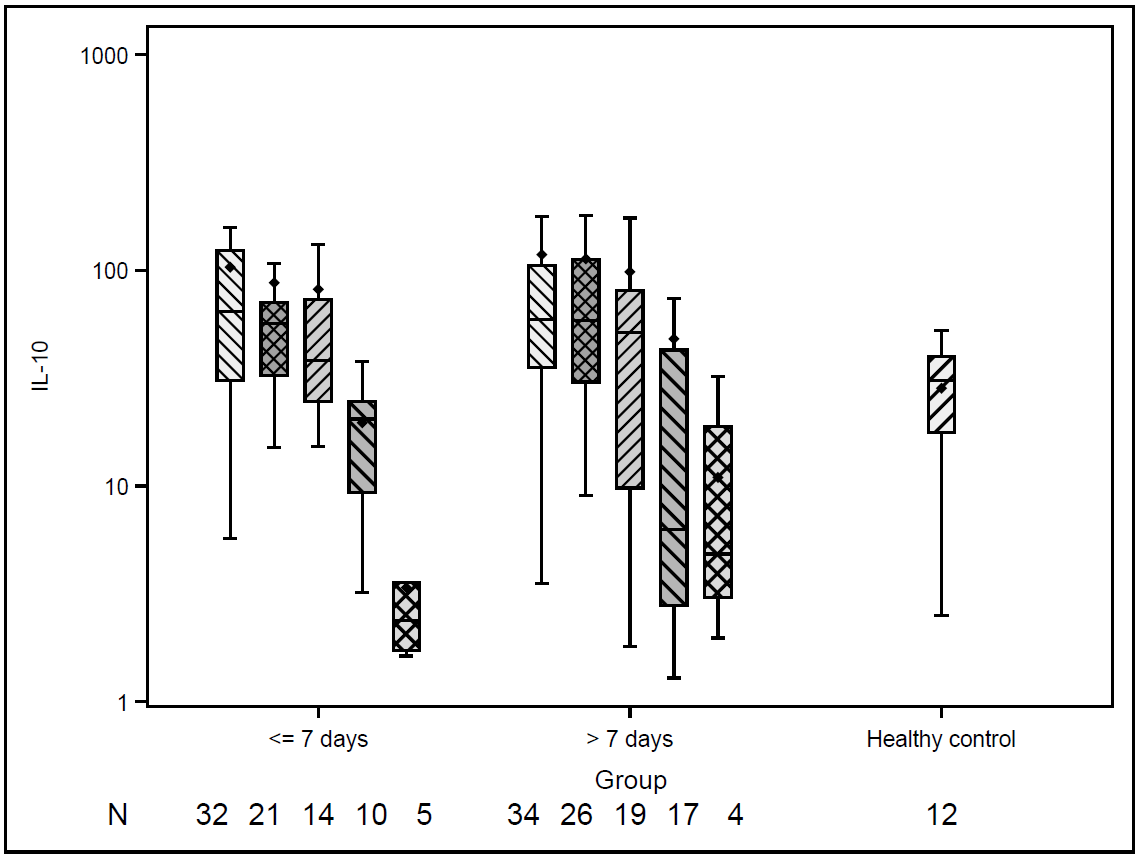 | 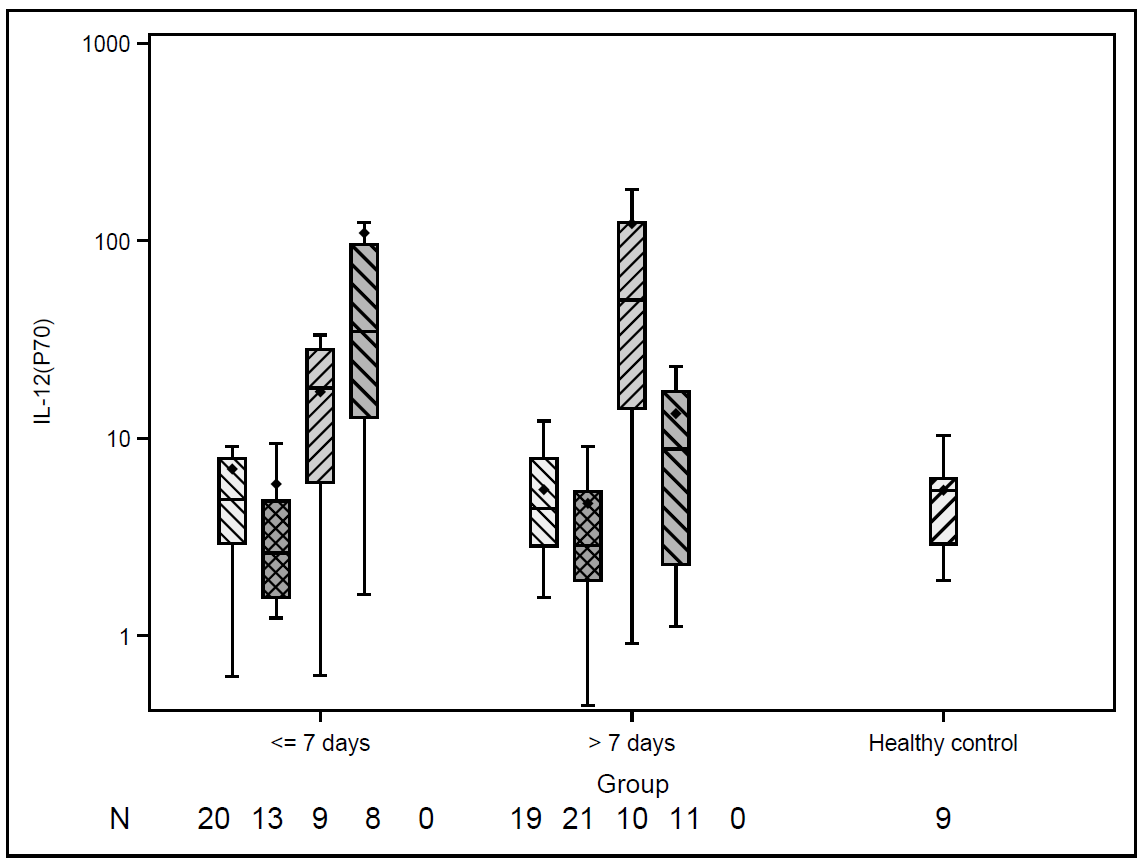 |
| P= 1.00 between the early and late treatment groups over time  P= 0.008 between early treatment group on D1 and healthy controls  P= 0.003 between the late treatment group on D1 and healthy controls | P= 1.00 between the early and late treatment groups over time  P= 0.98 between the early treatment group on D1 and healthy controls  P= 0.88 between the late treatment group on D1 and healthy controls |

| **IL-13** | **IL-17** |
| --- | --- |
| 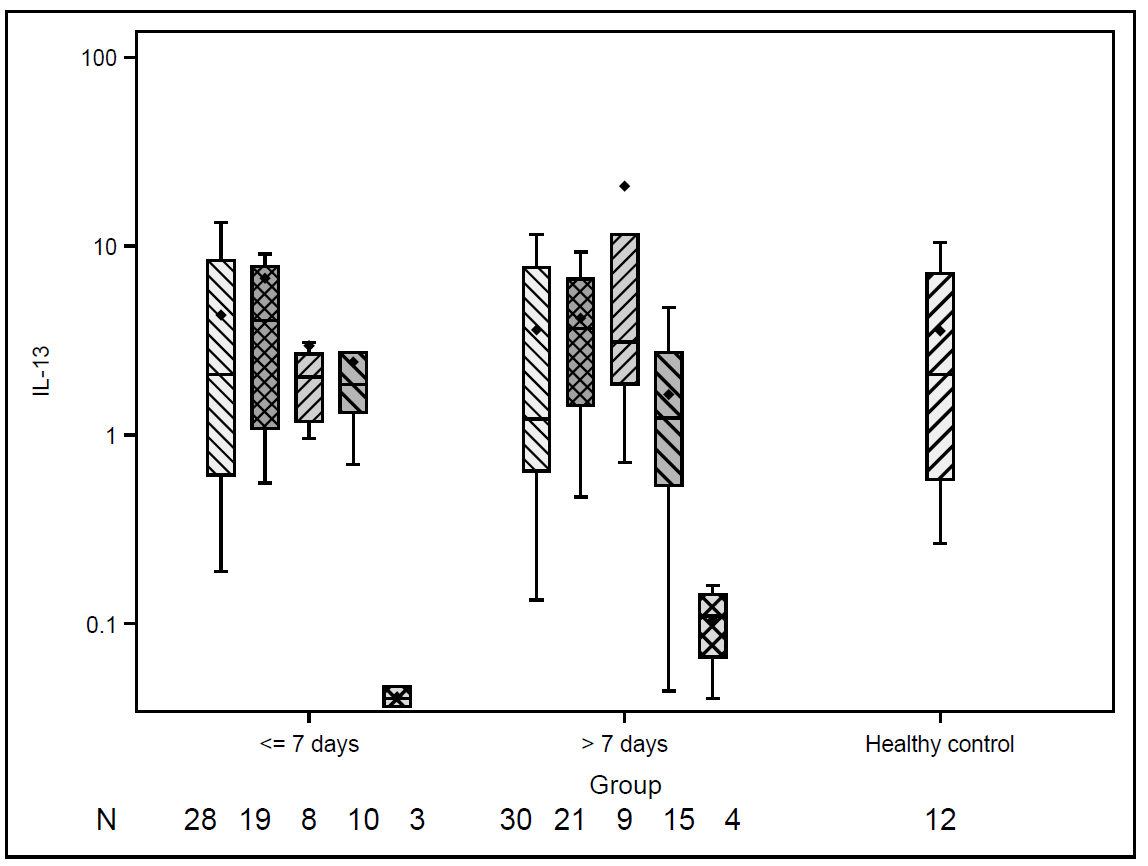 | 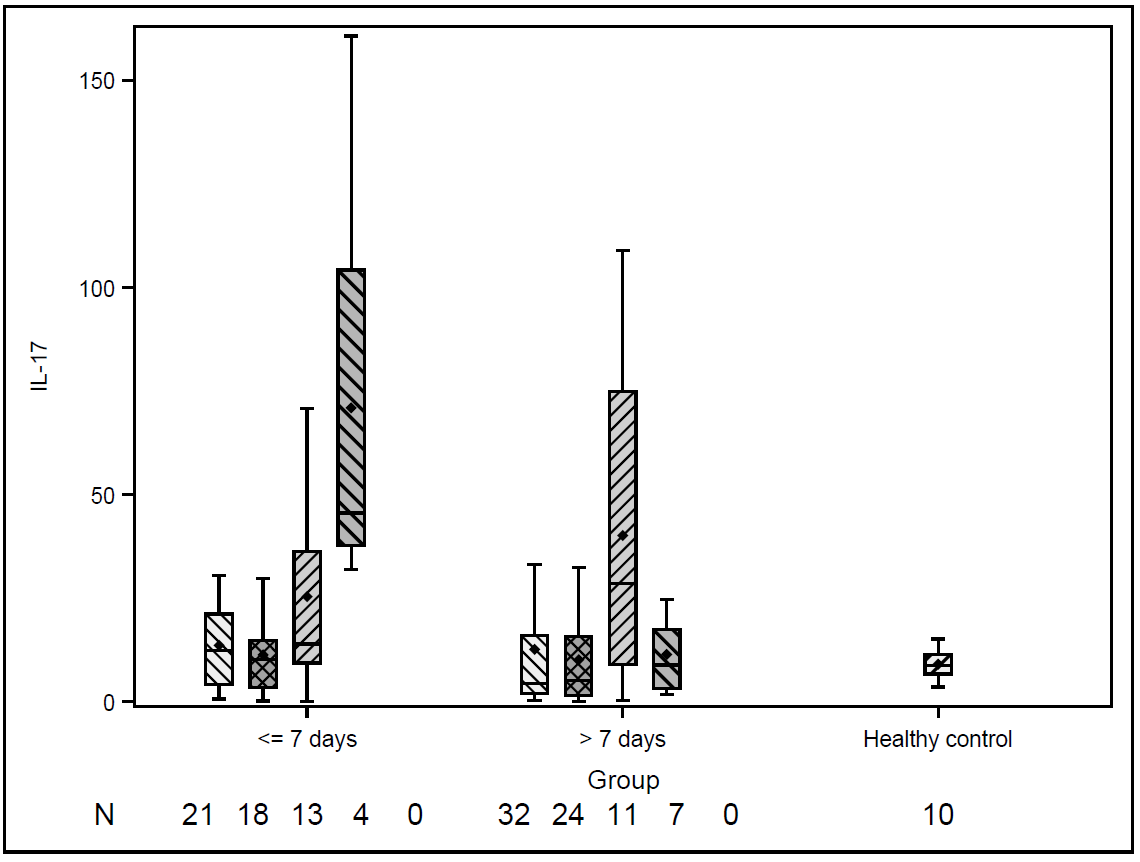 |
| P= 0.017 between the early and late treatment groups over time  P= 0.69 between the early treatment group on D1 and healthy controls  P= 0.96 between the late treatment group on D1 and healthy controls | P= 0.0001 between the early and late treatment groups over time  P= 0.39 between the early treatment group on D1 and healthy controls  P= 0.30 between the late treatment group on D1 and healthy controls |
| **MCP-1** | **MIP-1β** |
| 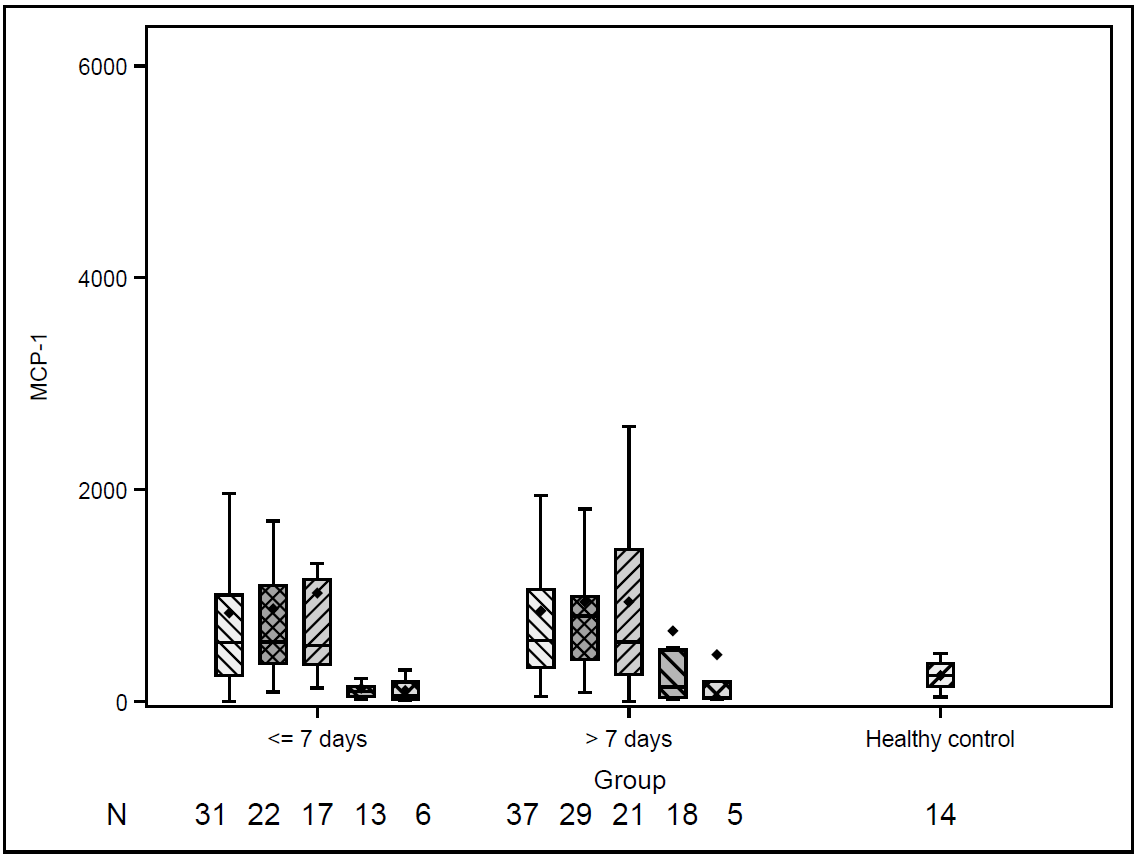 | 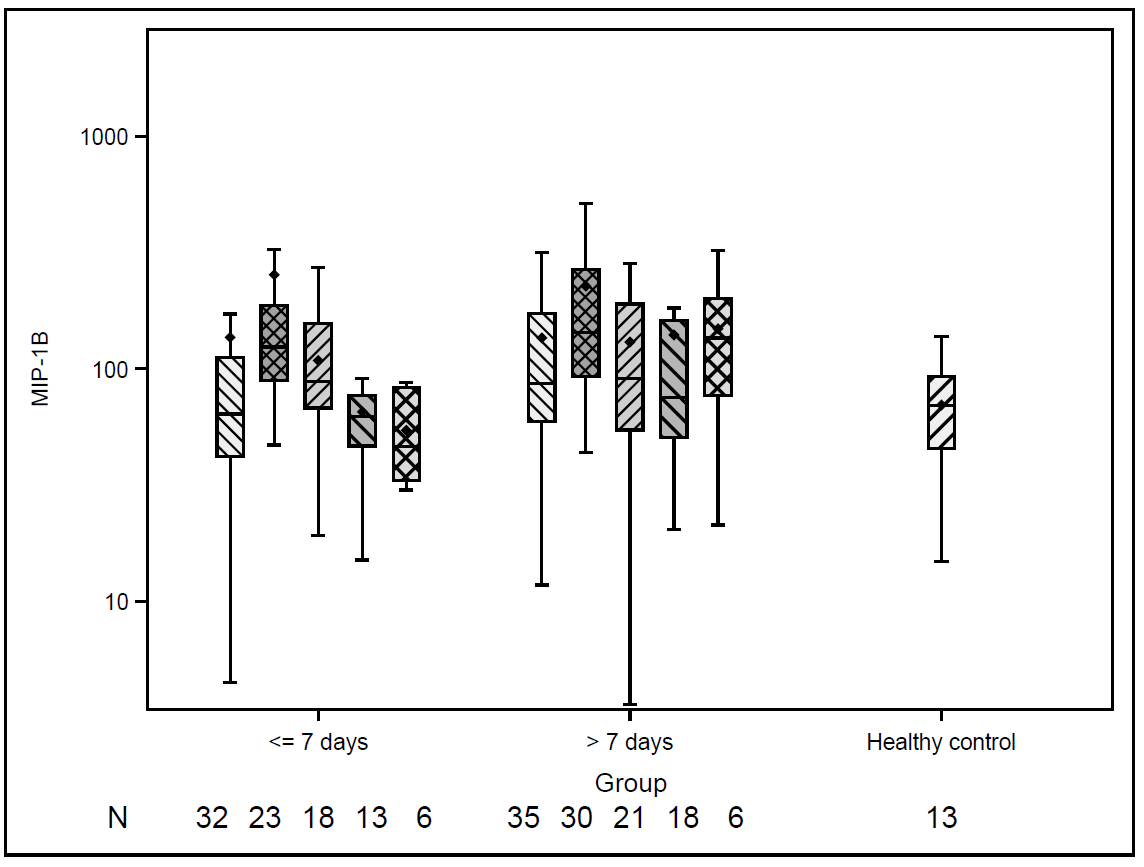 |
| P= 0.75 between the early and late treatment groups over time  P= 0.0050 between early treatment group on D1 and healthy controls  P= 0.0005 between late treatment group on D1 and healthy controls | P= 0.61 between the early and late treatment groups over time  P= 0.95 between the early treatment group on D1 and healthy controls  P= 0.12 between the late treatment group on D1 and healthy controls |
| **TNF-α** |  |
| 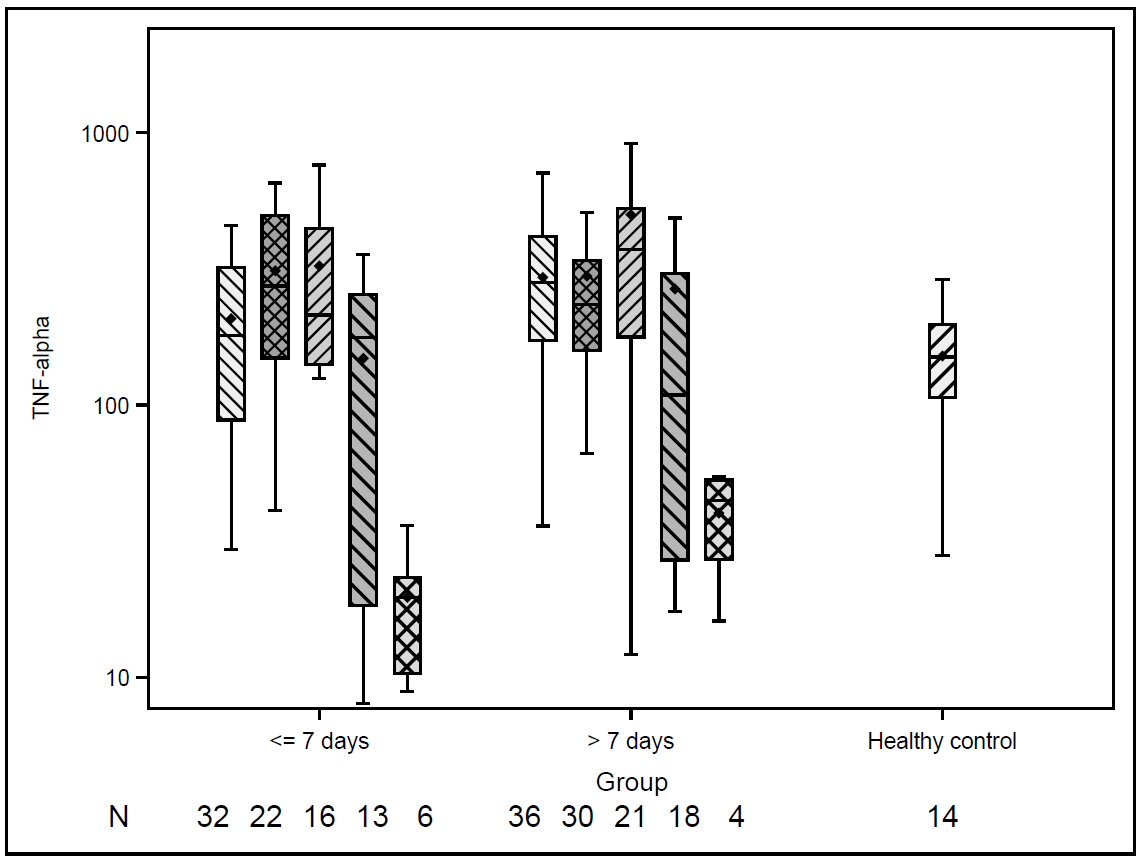 |  |
| P= 0.54 between the early and late treatment groups over time  P= 0.20 between the early treatment group on D1 and healthy controls  P= 0.0048 between late treatment group on D1 and healthy controls |  |
| G-CSF: granulocyte-colony stimulating factor; GM-CSF: granulocyte-macrophage colony-stimulating factor; IFN: interferon; IL: interleukin; MCP: Monocyte Chemo-attractant Protein; MIP: Macrophage inflammatory protein; TNF: tumor necrosis factor | |

**Figure S3:** Serial measurements for cytokines in survivors, non-survivors and healthy control. All cytokine levels were calculated based on mean fluorescent intensity and reported in pg/mL. We compared serial cytokine levels between survivors and non-survivors using a mixed linear model. We compared D1 values in both groups with those of healthy control using Mann–Whitney U test.

| 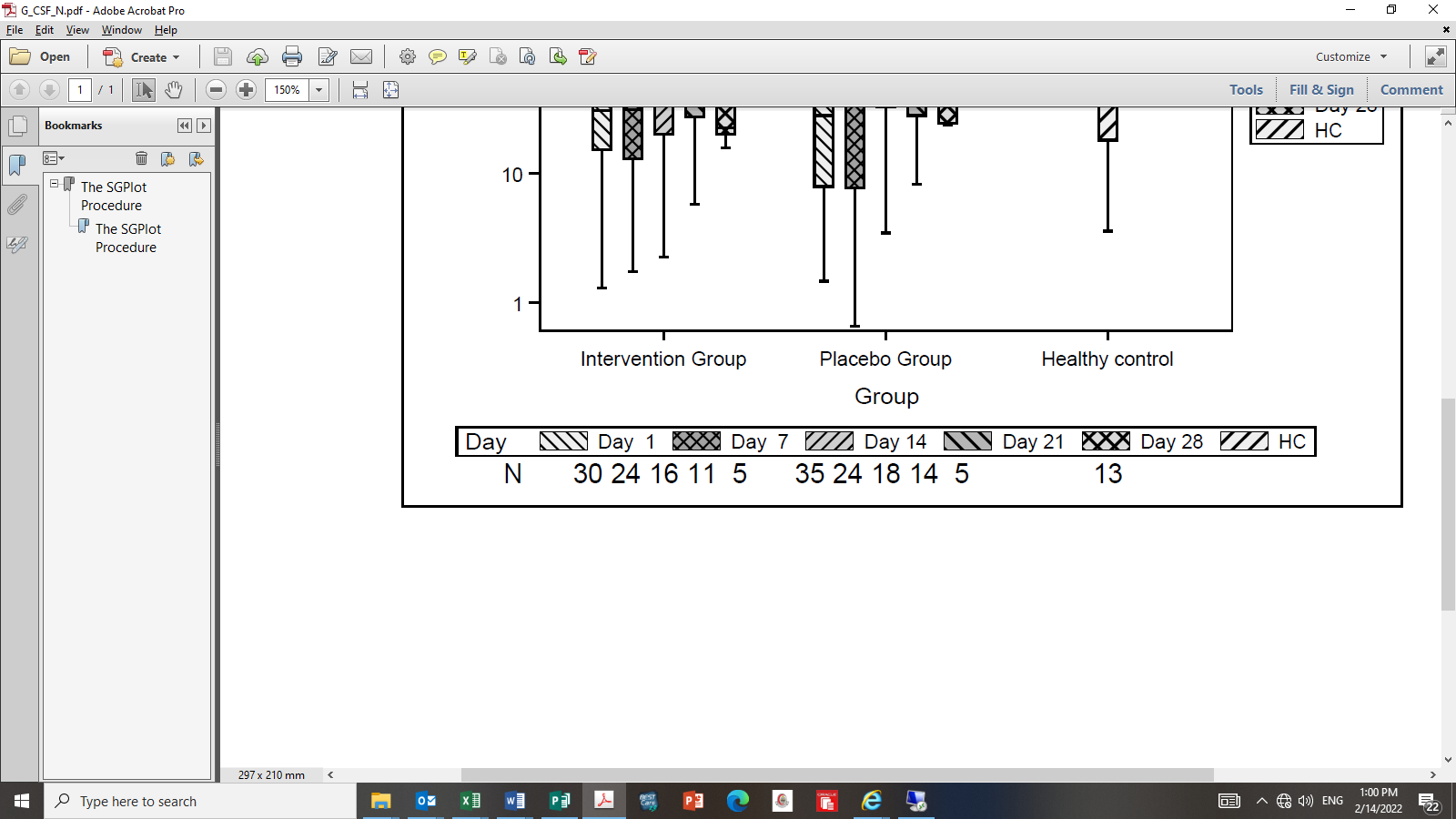 | |
| --- | --- |
| **G-CSF** | **GM-CSF** |
| 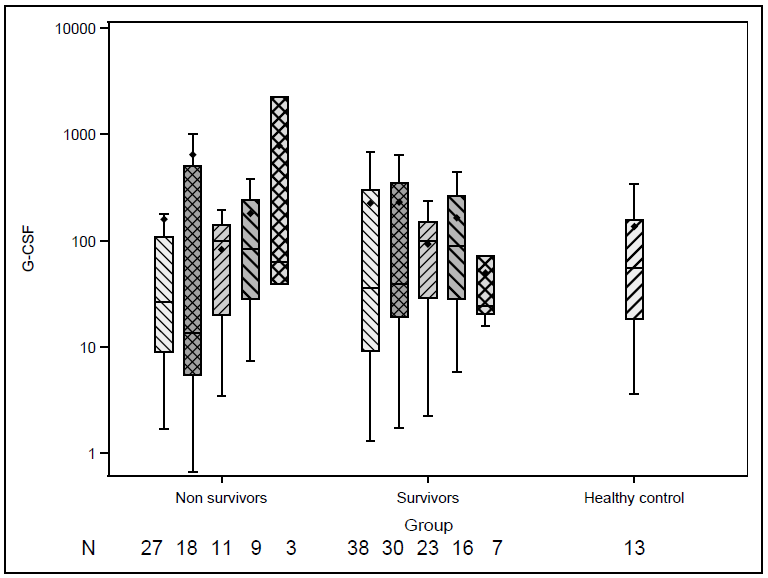 | 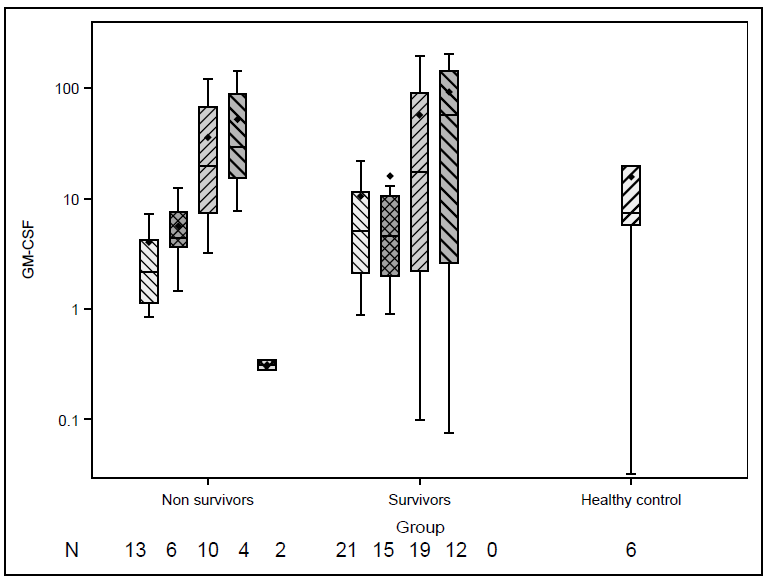 |
| P= 0.20 between the survivors and non-survivors over time  P= 0.85 between the survivors on D1 and healthy controls  P= 0.37 between the non-survivors on D1 and healthy controls | P= 0.84 between the survivors and non-survivors over time  P= 0.58 between the survivors on D1 and healthy controls  P= 0.13 between the non-survivors on D1 and healthy controls |
| **IFN-γ** | **IL-1β** |
| 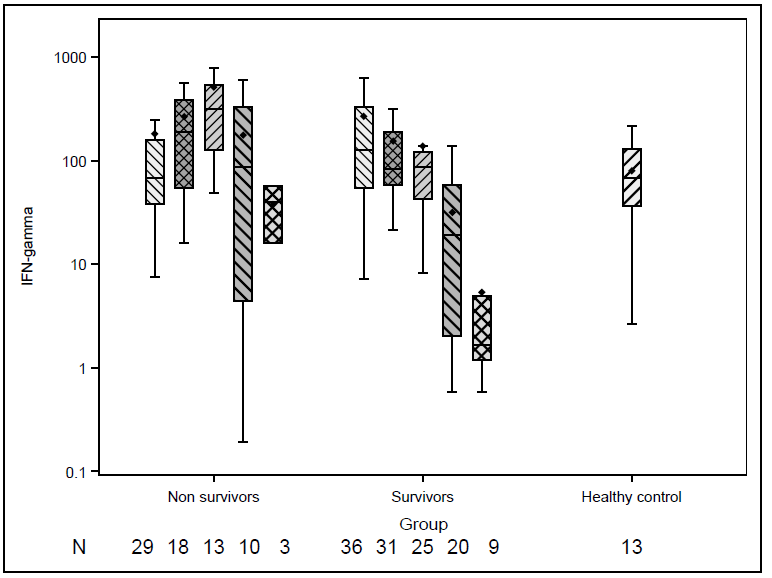 | 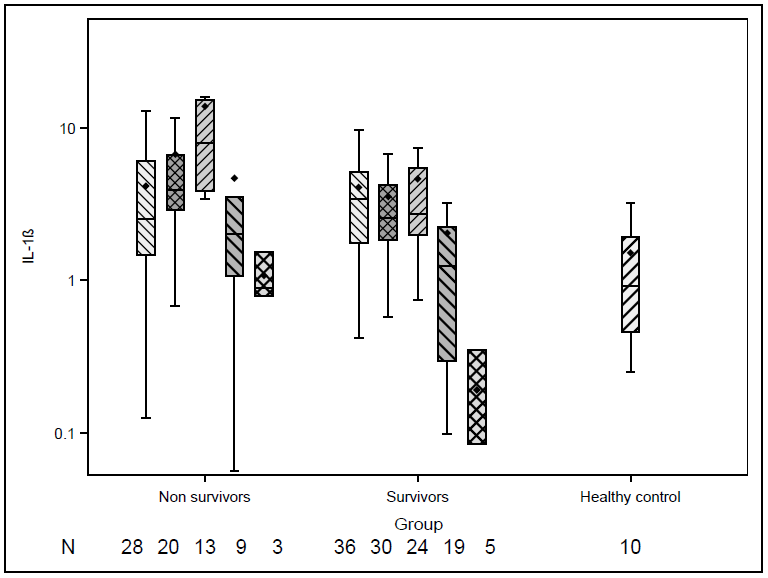 |
| P= 0.0097 between the survivors and non-survivors over time  P= 0.062 between the survivors on D1 and healthy controls  P= 0.45 between the non-survivors on D1 and healthy controls | P= 0.004 between the survivors and non-survivors over time  P= 0.0050 between the survivors on D1 and healthy controls  P= 0.025 between the non-survivors on D1 and healthy controls |
| **IL-2** | **IL-4** |
| 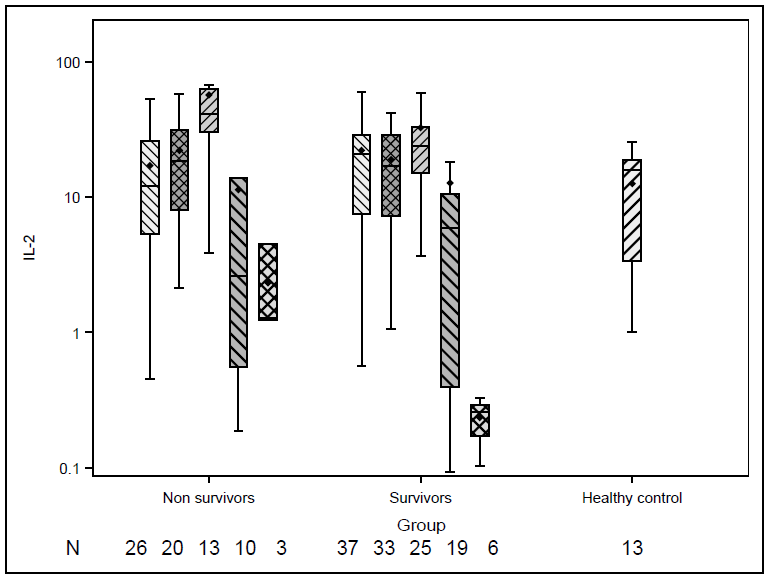 | 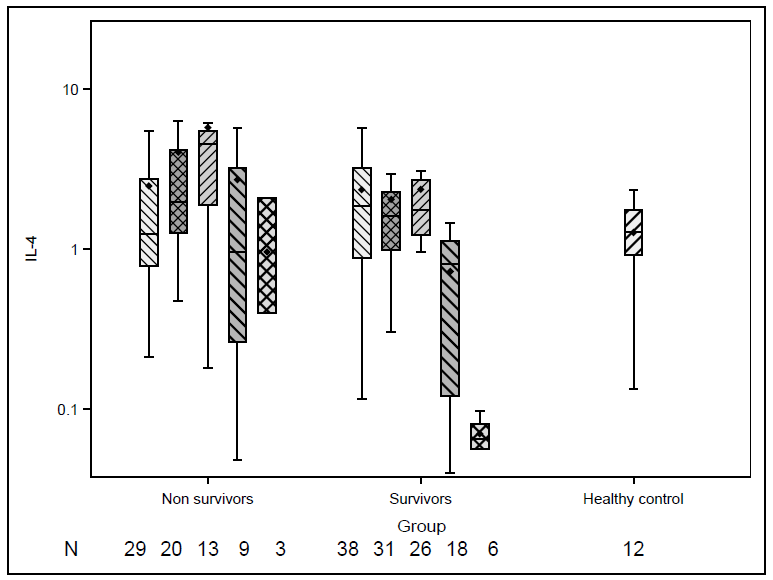 |
| P= 0.052 between the survivors and non-survivors over time  P= 0.11 between the survivors on D1 and healthy controls  P= 0.56 between the non-survivors on D1 and healthy controls | P= 0.11 between the survivors and non-survivors over time  P= 0.12 between the survivors on D1 and healthy controls  P= 0.40 between the non-survivors on D1 and healthy controls |

| **IL-5** | **IL-6** |
| --- | --- |
| 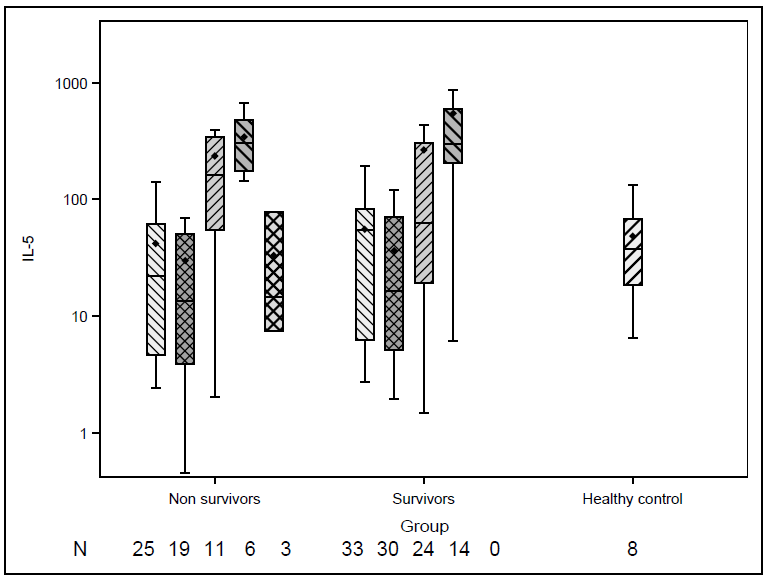 | 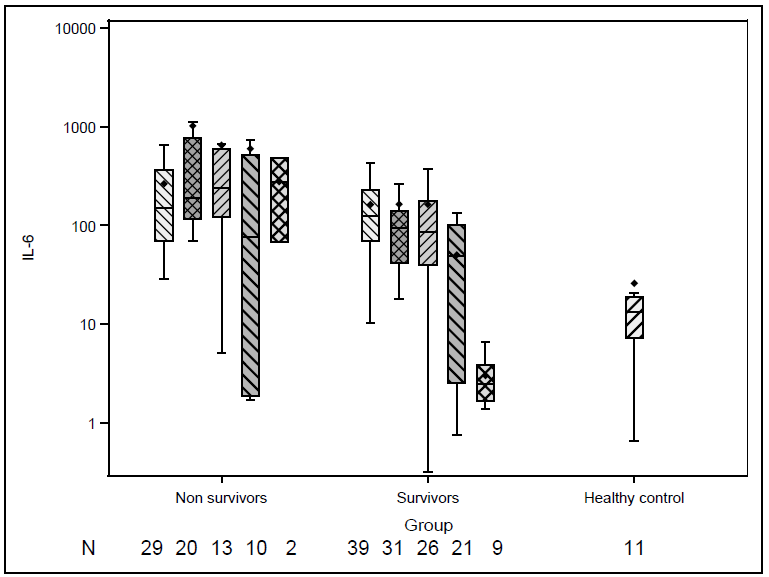 |
| P= 0.70 between the survivors and non-survivors over time  P= 0.94 between the survivors on D1 and healthy controls  P= 0.27 between the non-survivors on D1 and healthy controls | P= 0.24 between the survivors and non-survivors over time  P= <0.0001 between the survivors on D1 and healthy controls  P= <0.0001 between non-survivors on D1 and healthy controls |
| **IL-7** | **IL-8** |
| 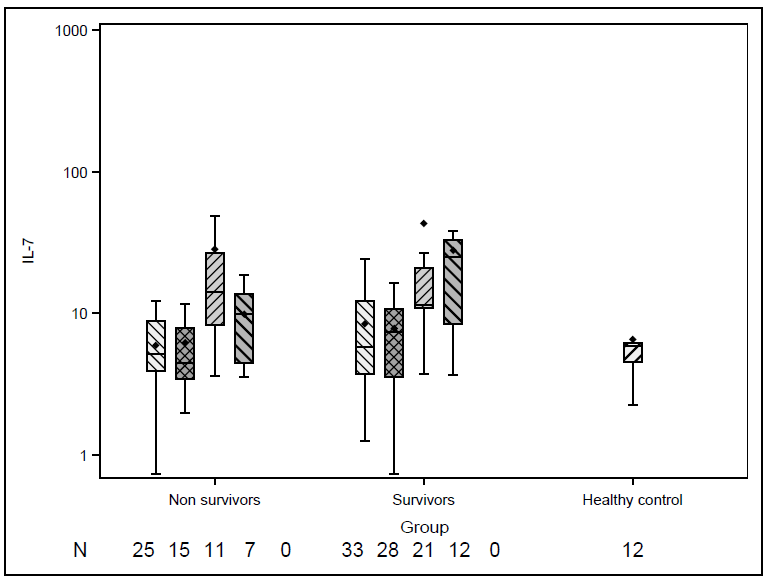 | 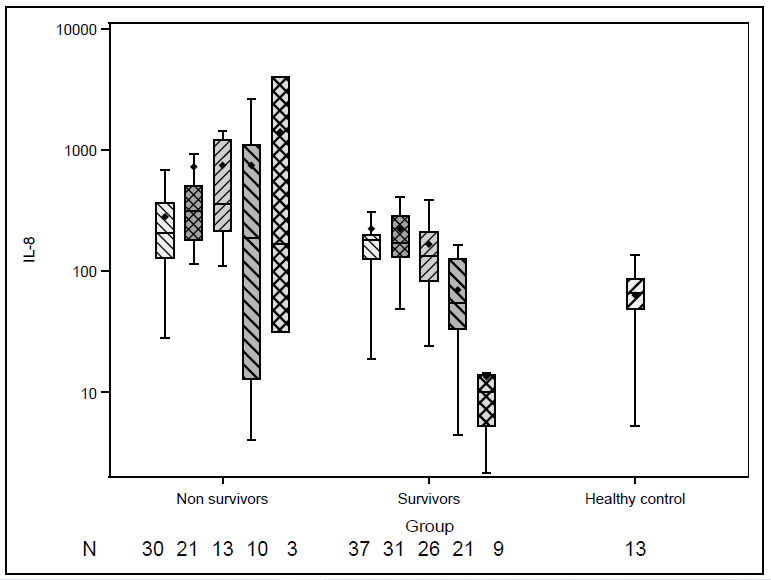 |
| P= 0.85 between the survivors and non-survivors over time  P= 0.87 between the survivors on D1 and healthy controls  P= 0.63 between the non-survivors on D1 and healthy controls | P= 0.016 between the survivors and non-survivors over time  P= 0.0002 between the survivors on D1 and healthy controls  P= <0.0001 between non-survivors on D1 and healthy controls |
| **IL-10** | **IL-12 (P70)** |
| 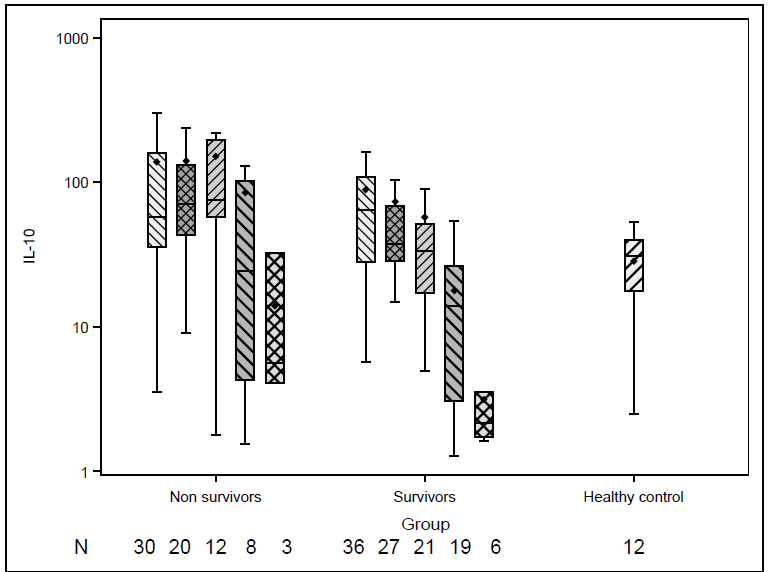 | 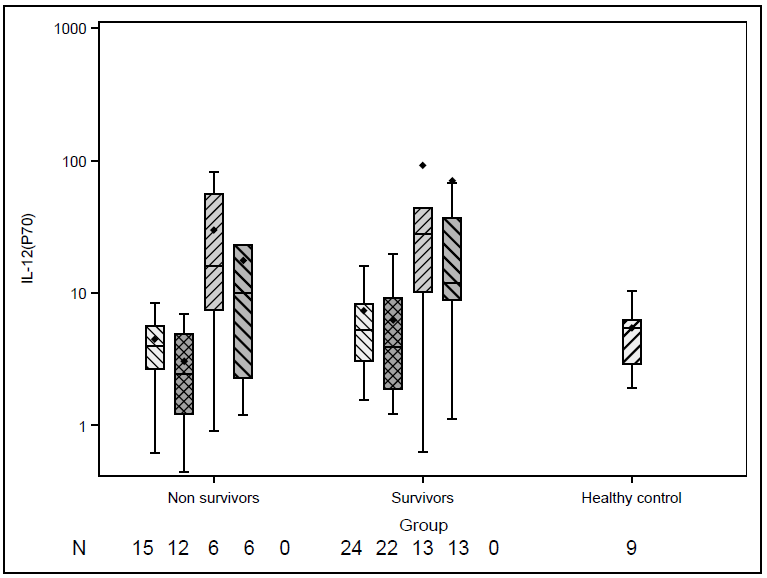 |
| P= 0.94 between the survivors and non-survivor over time  P= 0.006 between the survivors on D1 and healthy controls  P= 0.005 between the non-survivors on D1 and healthy controls | P= 0.41 between the survivors and non-survivors over time  P= 0.70 between the survivors on D1 and healthy controls  P= 0.47 between the non-survivors on D1 and healthy controls |

| **IL-13** | **IL-17** |
| --- | --- |
| 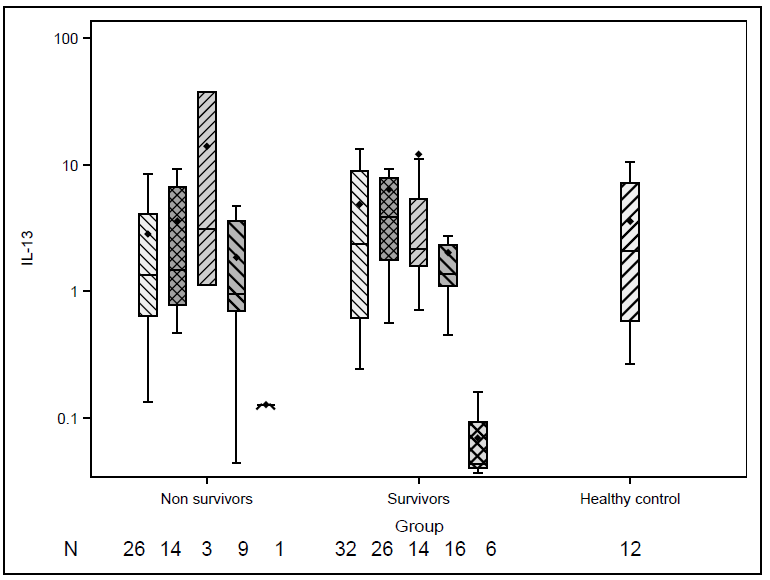 | 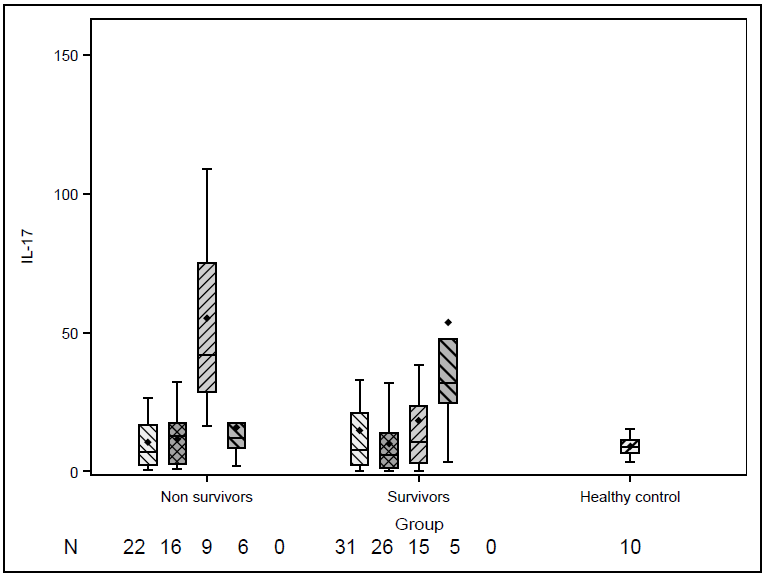 |
| P= 0.97 between the survivors and non-survivors over time  P= 0.47 between the survivors on D1 and healthy controls  P= 0.73 between the non-survivors on D1 and healthy controls | P= <.0001 between the survivors and non-survivors over time  P= 0.94 between the survivors on D1 and healthy controls  P= 0.64 between the non-survivors on D1 and healthy controls |
| **MCP-1** | **MIP-1β** |
| 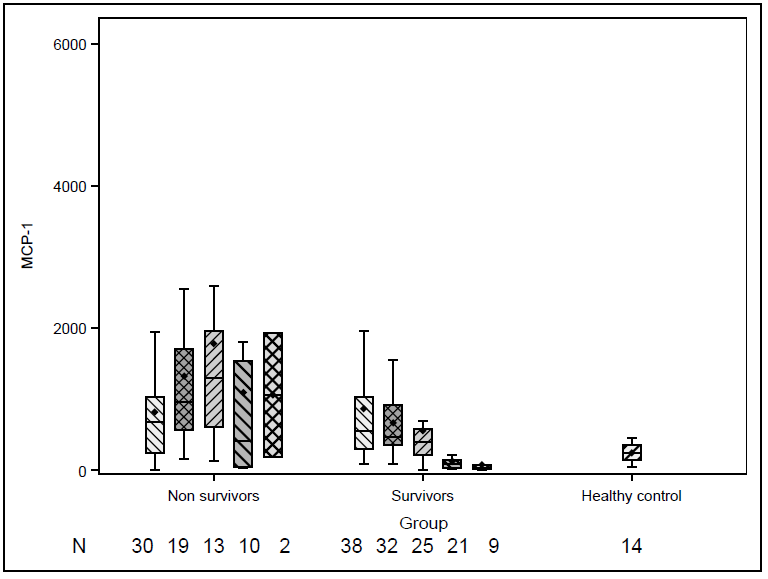 | 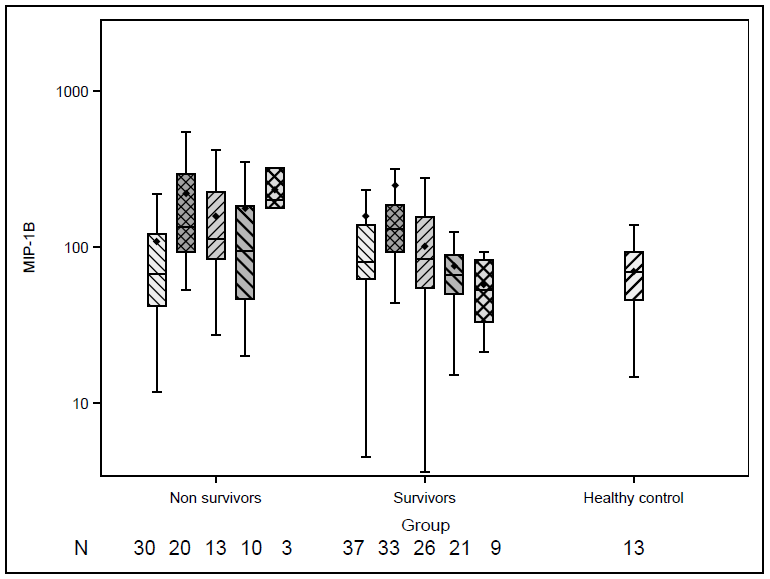 |
| P= 0.009 between the survivors and non-survivors over time  P= 0.0003 between the survivors on D1 and healthy controls  P= 0.008 between the non-survivors on D1 and healthy controls | P= 0.60 between the survivors and non-survivors over time  P= 0.26 between the survivors on D1 and healthy controls  P= 0.61 between the non-survivors on D1 and healthy controls |
| **TNF-α** |  |
| 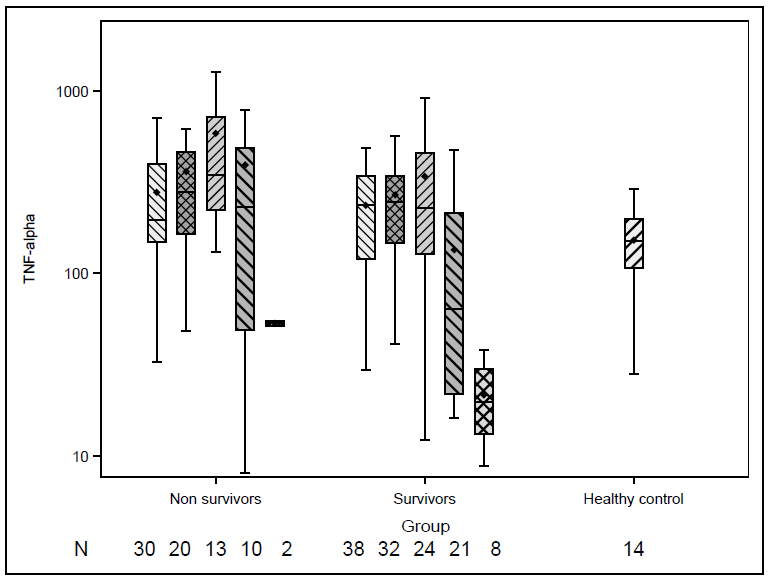 |  |
| P= 0.24 between the survivors and non-survivors over time  P= 0.039 between the survivors on D1 and healthy controls  P= 0.033 between the non-survivors on D1 and healthy controls |  |
| G-CSF: granulocyte-colony stimulating factor; GM-CSF: granulocyte-macrophage colony-stimulating factor; IFN: interferon; IL: interleukin; MCP: Monocyte Chemo-attractant Protein; MIP: Macrophage inflammatory protein; TNF: tumor necrosis factor | |

**Figure S4:** Exploratory analyses for defining the higher and lower levels of each of cytokines using the median or the median (Panel A) or lower tertile (33%) (Panel B) as cutoff points. The forest plot demonstrates the association of interferon-β1b and lopinavir-ritonavir treatment on 90-day mortality in patients with Middle East Respiratory Syndrome categorized into two subgroups of early and late treatment and according to higher and lower levels of each of the cytokines. The results are displayed as relative risks and 95% confidence intervals (CI). Additionally, p-values for the interactions are reported. Plasma cytokine concentrations are expressed in pg/ml.

| **Panel A:** Using the median as a cutoff point |
| --- |
| **** |
| **Panel B:** Using the lower tertile (33%) as a cutoff point |
| **** |
